# Effects of combined protein and probiotic supplementation on physical performance and body composition: a Bayesian multilevel meta-analysis of randomized controlled trials

**DOI:** 10.3389/fnut.2026.1865035

**Published:** 2026-06-17

**Authors:** Zhizhao Chang, Yiran Liu, Shiao Zhao, Xuda Zhang, Ni An

**Affiliations:** 1 Department of Physical Education and Research, Lanzhou University, Lanzhou, Gansu, China; 2 Faculty of Health Sciences and Sports, Macao Polytechnic University, Macau, Macao SAR, China

**Keywords:** body composition, meta-analysis, physical performance, probiotics, protein

## Abstract

**Background:**

Protein supplementation is widely used to support muscle adaptation. Probiotics may also improve nutrient use, gut health, and recovery. However, evidence on their combined effects remains inconsistent. This study examined the effects of protein-plus-probiotic supplementation on physical performance and body composition using Bayesian multilevel meta-analysis.

**Method:**

This review was prospectively registered in PROSPERO and conducted according to PRISMA. Randomized controlled trials in adults were identified through systematic database searches, with screening supported by ASReview and Covidence. Studies were included if they compared protein-plus-probiotic supplementation with placebo, protein alone, or other relevant controls, and reported outcomes on physical performance or body composition. Effect sizes were calculated as Hedges’ *g*. Bayesian multilevel models were used to account for dependent effect sizes within studies.

**Result:**

Twenty studies with 679 participants were included. The posterior estimate was positive for physical performance [Hedges’ *g*: 0.45, 95% HDI: 0.21 to 0.67], whereas the estimate for body composition was close to null [Hedges’ *g*: 0.02, 95% HDI: −0.14 to 0.20]. Signals of benefit were observed for muscle strength, muscular endurance, and lean mass, although these findings should be interpreted in light of outcome heterogeneity and study-level limitations.

**Conclusion:**

Current evidence suggests selective signals of benefit for protein-plus-probiotic supplementation. The intervention may be associated with better physical performance, particularly muscle strength and muscular endurance, whereas evidence for broad body-composition changes remains limited. For body composition, the most consistent signal was observed for lean mass or muscle mass. Further high-quality randomized trials are needed to clarify whether these signals are reproducible across probiotic strains, protein types, delivery forms, and populations.

**Systematic review registration:**

https://www.crd.york.ac.uk/PROSPERO/view/CRD420251165192, Registered at the international prospective register of systematic reviews (PROSPERO; CRD420251165192).

## Introduction

Protein and probiotics have both received increasing attention in sports nutrition and health promotion (1, 2). Protein is widely recognized as a key nutritional factor for muscle repair, muscle growth, exercise recovery, and the maintenance of favorable body composition (3–5). Probiotics have also emerged as a promising strategy because selected strains may support gut health, improve nutrient handling, and influence recovery related processes (2, 6, 7). Against this background, combining protein with probiotics has become an appealing idea. This approach may target both the nutritional substrate needed for muscle adaptation and the intestinal environment that supports nutrient use (8). However, although this concept is biologically plausible and practically attractive, the current evidence remains limited and sometimes inconsistent (6, 9, 10). Some studies have reported favorable effects on physical performance or body composition, whereas others have found small, uncertain, or null effects (6, 9, 10). These inconsistencies make it difficult to draw firm conclusions and highlight the need for a systematic review that can provide a more reliable synthesis of the available evidence.

Several biological mechanisms may explain why protein plus probiotic supplementation could influence physical performance and body composition (6, 11). Protein contributes directly to muscle adaptation because it provides essential amino acids that stimulate post-exercise muscle protein synthesis and support gains in lean mass and strength over time (3, 4, 11). Probiotics may act through different but related pathways. Selected strains can help maintain gut barrier integrity, regulate immune and inflammatory responses, and improve gastrointestinal function (6, 12). Some strains may also facilitate the digestion of dietary protein and increase the absorption or availability of amino acids and small peptides (10). When protein and probiotics are combined, these actions may work together (10). Protein provides the material needed for adaptation, while probiotics may help create a more favorable intestinal and metabolic environment for nutrient utilization and recovery (11). Taken together, these mechanisms provide a plausible biological basis for the potential benefits of protein plus probiotic supplementation. At the same time, they also suggest that the observed effects may depend on factors such as probiotic strain, delivery form, outcome indicator, and participant characteristics (6, 13).

In recent years, more studies and reviews have examined the effects of probiotics, and in some cases protein-plus-probiotic strategies, on exercise-related outcomes and body composition, although the findings have remained heterogeneous across strains, populations, and outcome measures (6, 9, 10). Some evidence has been encouraging. Recent reviews and position papers suggest that selected probiotic strains may benefit some muscle-related or recovery-related outcomes, although effects on body-composition indicators are not consistently observed across studies (6). Individual trials have also reported positive findings for combined supplementation. For example, one study found that combining *Lactiplantibacillus plantarum* TWK10 with pea protein and resistance training was associated with greater amino acid uptake and more favorable adaptations in adults than pea protein alone (14). Another trial reported that *Bacillus coagulans* GBI 30, 6,086 combined with protein tended to reduce exercise induced muscle damage, improve recovery, and help maintain performance after damaging exercise (10). Together, these findings suggest that protein plus probiotic supplementation may have selective benefits, particularly for muscle-related outcomes and some aspects of physical performance. They also support the view that the gut muscle axis may have practical relevance in intervention settings rather than being only a theoretical concept.

However, these potential benefits have not been confirmed consistently across all studies or all outcomes (15, 16). The same meta-analysis that reported positive effects on muscle mass and strength also found no significant improvement in total lean mass (17). Reviews in athletes and physically active populations have likewise described probiotic effects on performance as mixed rather than uniformly positive (15, 16). A likely reason is the substantial heterogeneity across studies (18). The available trials differ in probiotic strain, protein source, delivery matrix, intervention duration, training background, and outcome assessment (6, 18, 19). These differences may strongly influence whether a positive effect can be detected (6, 18). In some cases, the intervention may be more relevant to recovery and muscle-related adaptation than to broader changes in body composition (6, 10, 17). In other cases, the duration may be too short, the participants may already have adequate nutritional status, or the selected outcomes may not be sensitive enough to capture change. In addition, current evidence suggests that probiotic effects are strain specific, and some work has indicated that delivery matrix may also influence probiotic efficacy (6, 19). Therefore, because of the inconsistency across findings, participant characteristics, and supplementation strategies, the current evidence is still insufficient to support a firm overall conclusion about the efficacy of protein plus probiotic supplementation.

Previous work from our group used a Bayesian multilevel framework to synthesize randomized controlled trials of protein supplementation in athletes, with a focus on athletic performance, post-exercise recovery, protein source, timing, dose, and energy matching (20). The present review addresses a different intervention question. It focuses specifically on protein-plus-probiotic co-supplementation in adults, includes physical performance and body composition as core outcome domains, and examines probiotic-related features such as strain composition, specific strain, and delivery form. Where trial reports met both eligibility frameworks, they were reassessed according to the current PICOS, and the relevant effect sizes were re-extracted or recalculated for the present outcomes, comparator categories, and effect-direction rules. Based on the current but inconsistent literature, we hypothesized that protein plus probiotic supplementation may improve physical performance and body composition, but that these benefits would be selective rather than universal across all outcomes.

## Methods

This study was prospectively registered with PROSPERO (CRD 420251165192) and followed the PRISMA reporting guidelines (21). The systematic review was conducted alongside a Bayesian meta-analysis using the following tools: ASReview (22) and Covidence for study management, GRADEprofiler for evidence assessment, R version 4.5.1 for statistical analysis, and GetData Graph Digitizer for data extraction from figures.

### Literature search

A systematic search was carried out in Web of Science, PubMed, EMBASE, the Cochrane Library, Scopus, and EBSCO. The database search and eligibility assessment were restricted to studies published in English. The strategy combined free-text terms and controlled vocabulary related to protein supplementation, probiotics, combined or adjunct use, exercise performance, body composition, and metabolism. Two reviewers developed the search collaboratively and applied it in parallel. In addition, the reference lists of all eligible articles were hand-searched to capture studies that might have been missed. Any uncertainties encountered during the process were resolved by a third reviewer. Detailed search strings for each database are provided in Supplementary File S1.

### Eligibility criteria

Eligibility was determined using the PICOS framework. The inclusion criteria were as follows:

Population (P): Adults aged 18 years or older were eligible, with no restriction on sex or training status.

Intervention (I): Eligible interventions involved co-administration of a dietary protein source with a probiotic-containing preparation within the same feeding occasion or daily regimen. Both supplement-type formulations and food-based delivery matrices were eligible.

Comparator (C): Appropriate controls included protein alone, placebo, or another non-protein comparator judged to be relevant to the intervention question.

Outcomes (O): Trials had to report at least one physical performance outcome or body composition outcome and provide sufficient information to compute effect sizes.

### Study selection

All titles and abstracts were screened with support from ASReview, a machine learning system that uses active learning to prioritize records so that screening proceeds in a rigorous and reproducible way (22). The model was initialized with 10 seed records, including five records labeled as relevant and five records labeled as irrelevant. Relevant seed records were selected from studies known to meet the eligibility criteria or identified during pilot screening. Irrelevant seed records were selected from records that were clearly outside the scope of the review, such as records with an ineligible population, intervention, study design, or outcome.

After initialization, the model was updated after each reviewer decision and presented the next record with the highest predicted relevance. Screening was stopped after 200 consecutive records had been judged as irrelevant. After ASReview-assisted screening, we conducted a post-hoc audit of 200 unscreened records using the same title-and-abstract eligibility criteria. To further reduce the risk of missing eligible trials, the reference lists of included studies and relevant reviews were also checked manually.

In the next stage, two reviewers (Y. L. and Z. C.) carried out full-text screening in Covidence in line with PRISMA 2020 reporting guidance (21). Screening by at least two independent reviewers with disagreements resolved by discussion or adjudication by a third reviewer is consistent with established methodological standards. A pre-specified Extraction Form version 1.0 was used to evaluate all eligible full texts, and any disagreements were settled by a third reviewer.

### Data extraction

Two reviewers (Y. L. and Z. C.) independently extracted study characteristics and numerical outcome data, including first author, publication year, sample size, sex, mean age, training status, intervention and comparator details, intervention duration, and prespecified outcomes. Extracted datasets were cross-checked, and disagreements were resolved through discussion with a third reviewer. Because a previous review from our group addressed protein supplementation in athletes, we checked whether eligible reports had appeared in that review (20). Reports were included in the present review only when they met the current protein-plus-probiotic PICOS, and no pooled estimates or aggregate results from the previous review were reused.

For continuous outcomes, we preferentially extracted change-from-baseline means and standard deviations for each group. When change scores were not reported, they were reconstructed from baseline and post-intervention summaries using established methods. Mean change was defined as the post-intervention mean minus the baseline mean, and the standard deviation of the change score was derived from the baseline and post-intervention standard deviations while accounting for the within-group correlation between repeated measurements (23). When this correlation was unavailable from the trial report or study authors, a fixed value of 0.50 was imputed as a pragmatic assumption, consistent with published meta-analytic practice for handling missing change-score variance information. Published reviews also emphasize that such assumptions should be reported clearly (24).

The standardized mean difference was then calculated from the between-group difference in mean change and converted to Hedges’ g using the usual small-sample correction, in line with methodological work on pretest-posttest-control group designs and the original literature on small-sample bias correction (25).

When required, additional statistics were derived from standard errors, confidence intervals, t statistics, or exact *p*-values (26).

If key numerical data remained unavailable, we contacted study authors. When results were presented only in figures, values were digitized using GetData Graph Digitizer, consistent with published evidence that software-assisted graphical data extraction can be reliable and valid when performed carefully (27).


$$\Delta =M_{\text{post}}-M_{\mathrm{pre}}$$

$$SD_{\Delta }=\sqrt{SD_{\mathrm{pre}}^{2}+SD_{\text{post}}^{2}-2r\times SD_{\mathrm{pre}}\times SD_{\text{post}}}$$

### Risk of bias assessment

Risk of bias was assessed using the revised Cochrane risk-of-bias tool for randomized trials (RoB 2) (28). Two authors independently assessed each included trial across five domains: randomization process, deviations from intended interventions, missing outcome data, measurement of the outcome, and selection of the reported result. Domain-level and overall judgments were rated as low risk, some concerns, or high risk according to the RoB 2 signaling questions and algorithm. Disagreements were resolved through discussion. The final judgments were recorded in Excel and visualized in R version 4.5.1 using the robvis package.

### Statistical analysis

Effect sizes were synthesized using Bayesian multilevel meta-analytic models implemented in the brms package (29). The primary effect-size metric was Hedges’ *g*, a small-sample corrected standardized mean difference (25). Where applicable, effect sizes were derived from between-group differences in pre-post change scores, and the direction of effects was harmonized such that positive values indicated a favorable effect of the combined protein-plus-probiotic intervention relative to the comparator.

Because several studies contributed multiple related effect sizes, the analyses explicitly accounted for effect-size dependence by specifying hierarchical random effects at the study level and the within-study effect-size level. This multilevel structure allowed correlated outcomes from the same trial to be retained without treating them as statistically independent observations (25). Physical performance was first analyzed as a broad domain because the included outcomes all represented exercise-related functional capacity and were coded in a common favorable direction using Hedges’ *g*. This domain-level model was used to provide an overall directional summary of the evidence and to avoid selecting only one performance outcome from each trial. However, this approach does not assume that muscle strength, muscular endurance, cardiorespiratory endurance, functional performance, and power/anaerobic performance measure the same physiological construct or respond similarly to supplementation. Therefore, the global physical-performance estimate was interpreted cautiously, and the outcome-indicator models were used as the primary basis for outcome-specific interpretation.

Bayesian multilevel models were fitted in brms. For the null model, the pooled effect was estimated using the model yi|se(sei) ~ 1 + (1|Authors) + (1|Authors:studyID), separately for physical performance and body composition. The intercept represented the overall pooled Hedges’ *g*. A weakly informative Normal (0, 0.5) prior was used for the pooled effect, and Exponential (Rate parameter is 2) priors were used for the study-level and within-study standard deviation parameters. For moderator models, zero-centered weakly informative priors were used for fixed effects, and weakly regularizing priors were used for variance components. The exact model syntax and prior specifications are provided in the analysis code.

Bayes factors for fixed-effect parameters were calculated against the point null value of zero using bayestestR:bayesfactor_parameters (30), based on the prior and posterior distributions of the corresponding parameters. These Bayes factors were treated as parameter-level indices of evidence against the point null for individual fixed-effect estimates and were not interpreted as formal model-comparison Bayes factors based on marginal likelihood estimation. Because no formal prior-sensitivity analysis was conducted, Bayes factors were used only as supplementary descriptors. The primary interpretation was based on posterior means, 95% HDIs, model convergence diagnostics, heterogeneity estimates, and consistency across related estimates. Heterogeneity was summarized using posterior tau parameters at the between-study and within-study levels. In addition, Decision Inconsistency (DI) and Across-Studies Inconsistency (ASI) indices were calculated using the metainc package (31).

We fitted nine models separately for the physical performance and body composition data sets:

(1) Null model to estimate the overall effect for each full data set. Separate models were fitted for the complete PP and BC data sets, respectively.

(2) Control model. This model separated protein plus probiotic versus protein alone from protein plus probiotic versus placebo or another non-protein comparator so that the added value question could be examined more directly.

(3) Outcome indicator model. This model classified endpoints within each domain to avoid combining substantively different indicators and to identify which outcomes showed the most consistent patterns.

Exploratory models. Additional models examined population, supplement form, probiotic type, probiotic strain, protein type, and funding. These analyses were retained for completeness, but they were not used as the main basis for the principal conclusions.

(4) Supplement form model (Capsule; Powder; Yogurt [fermented dairy]; Drink; Combination [capsule + powder]). This model classified the delivery form of the interventions.

(5) Probiotic type model (Single-strain; Multi-strain blend). This model compared broad probiotic preparation formats.

(6) Probiotic strain model (*B. coagulans* GBI-30, 6,086; *Bacillus coagulans* Unique IS-2; *Bacillus clausii* UBBC-07; *Weizmannia coagulans* BC99; *L. plantarum* TWK10; *L. helveticus*; *Lactobacillus casei* CNCM-1518; *Lactobacillus casei* DK211). This model examined strain-specific associations where data were sufficient.

(7) Protein type model (whey, casein, casein + whey, pea protein, pea+ rice protein, and spirulina, where data were sufficient). This model grouped protein sources into broad analytical categories.

(8) Population model (Adults; Athletes; Elderly; Overweight/Obesity; Clinical-Rehabilitation). This model was used to examine whether population characteristics contributed to between-study variation.

(9) Funding model (Funded vs. Unfunded). This model examined whether funding status was associated with differences in estimated effects.

### Outcome classification and direction of effects

Outcomes were classified before synthesis into two broad domains: physical performance and body composition. Physical performance outcomes were defined as objective or test-based measures of exercise-related function, including muscle strength, muscular endurance, cardiorespiratory endurance, functional performance, and power/anaerobic performance. Body composition outcomes were defined as measures of lean mass or muscle mass, adiposity, body mass, or body-size indicators. Within each domain, outcomes were further grouped into prespecified indicators according to conceptual similarity, measurement purpose, and the direction in which the outcome represented benefit. Outcome data were extracted according to the measurement methods reported in the original trials, including performance tests and body-composition assessment methods. Before analysis, effect directions were harmonized so that positive Hedges’ g values consistently indicated a favorable effect of protein-plus-probiotic supplementation. For outcomes where lower values indicated better status, such as fat mass, body fat percentage, or time-based performance tests, the sign of the effect size was reversed before synthesis.

### Publication bias

Potential publication bias was assessed using the PublicationBias R package (32, 33). We used significance funnel plots and s values for selective publication under favor_positive = TRUE. We also conducted Egger type regression with the metafor package as an exploratory diagnostic for small study effects (34). These procedures were interpreted cautiously because the number of studies was limited, the outcomes were heterogeneous, and non-significant findings do not rule out reporting bias.

### Certainty of evidence

Certainty of evidence was assessed using the GRADE approach (35). Because the included studies were randomized controlled trials, the initial certainty was rated as high and could be rated down across five domains: risk of bias, inconsistency, imprecision, indirectness, and publication bias. GRADE was applied as an outcome-level structured judgment rather than as an algorithmic scoring system.

Risk of bias judgments were informed by the RoB 2 assessment and were considered at the outcome level. The RoB 2 categories of low risk, some concerns, and high risk were used; no separate “moderate risk” category was applied. Evidence was rated down when the limitations of the contributing studies were judged serious enough to reduce confidence in the estimate, rather than because a specific number of studies had some concerns.

For inconsistency, we considered the direction and magnitude of effects across studies, overlap of interval estimates, and heterogeneity from the Bayesian multilevel models. For imprecision, we used the 95% HDI from the primary Bayesian model as the interval estimate for the pooled effect, because all main analyses were Bayesian. Imprecision was judged by whether the HDI was compatible with both no meaningful effect and a potentially important benefit, together with the number of studies and participants. Indirectness and publication bias were assessed according to standard GRADE considerations. The final certainty rating for each key outcome was summarized as high, moderate, low, or very low.

## Results

Detailed numerical estimates for the exploratory models are provided in Supplementary File S4, and the corresponding posterior summary plots and Bayes factor plots are provided in Supplementary File S5. Because these analyses were exploratory and hypothesis-generating, they were not used as the main basis for the principal conclusions.

### Literature selection

Database searches conducted in March 2026 yielded 6,946 records from Cochrane (284), EBSCO (129), PubMed (3,580), Embase (463), Web of Science (305), and Scopus (2,185). After removing duplicates, 5,871 unique records remained and were uploaded to ASReview. The ASReview model was initialized with five relevant and five irrelevant seed records. Using active-learning prioritization, 372 titles and abstracts were screened by human reviewers. Screening was stopped after 200 consecutive records had been judged as irrelevant, and 42 reports were taken forward for full-text assessment. The post-hoc audit examined 200 unscreened records, and no record was judged potentially relevant at the title-and-abstract level. Twenty-five reports were excluded at this stage for wrong outcomes in 2 records, wrong study design in 16 records, and wrong intervention in 7 records. No exclusions were due to population or language. Seventeen studies met the eligibility criteria.

**Figure 1:**
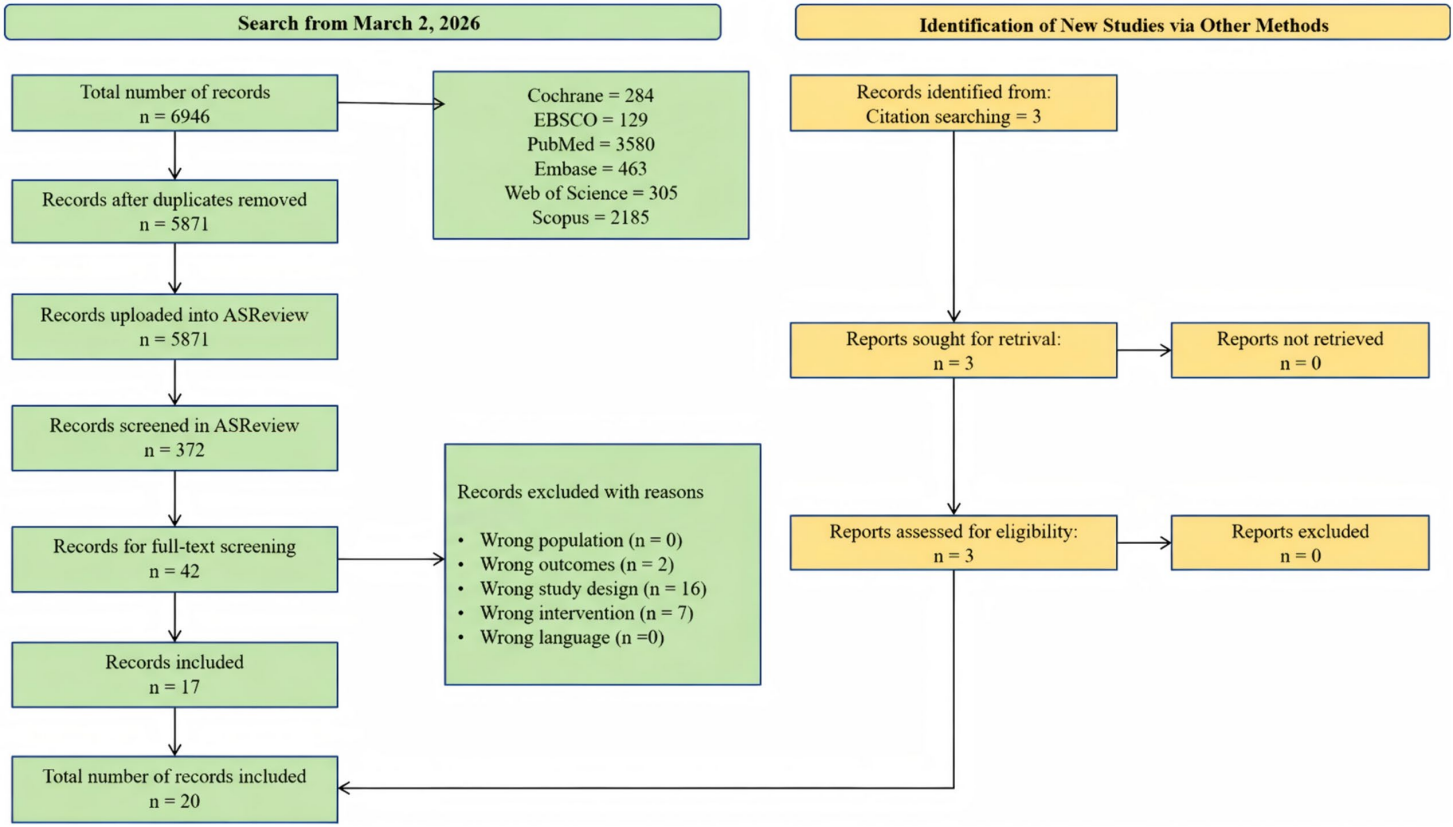
PRISMA flow diagram of literature screening.

To complement database searching, citation searching identified 3 additional records. All were retrieved and assessed at full text and none was excluded. The final evidence set included 20 studies (Figure 1).

### Characteristics of literature

Across 20 randomized controlled trials, a total of 679 participants were enrolled. Regarding population characteristics, most trials enrolled healthy adults: Adults, 11 trials (10, 14, 36–44). Athletic cohorts were represented by 5 trials (45–49). Additional subgroups included Elderly, 1 trial (50), Overweight, 2 trials (51, 52), and Clinical-Rehab, 1 trial (53). Formulations and delivery vehicles for the probiotic component were as follows: yogurt, 6 trials (36, 38, 43, 44, 50, 52), powder, 5 trials (14, 37, 40–42), drink, 5 trials (39, 45, 46, 51, 53), combination of capsule plus powder, 3 trials (10, 47, 49), and capsule, 1 trial (48). All trials reported a delivery form. By probiotic strain composition, monostrain products were used in 8 trials (14, 37–42, 47), multistrain products in 5 trials (10, 48, 49, 52, 53), and strain composition was not reported in 7 trials (36, 43–46, 50, 51). With respect to protein source, animal-based proteins were used in 17 trials (10, 36–44, 46, 47, 49–53), plant proteins in 2 trials (14, 45), and spirulina in 1 trial (48). Details of literature characteristics can be found in Supplementary File S2.

### Risk of bias assessment

**Figure 2:**
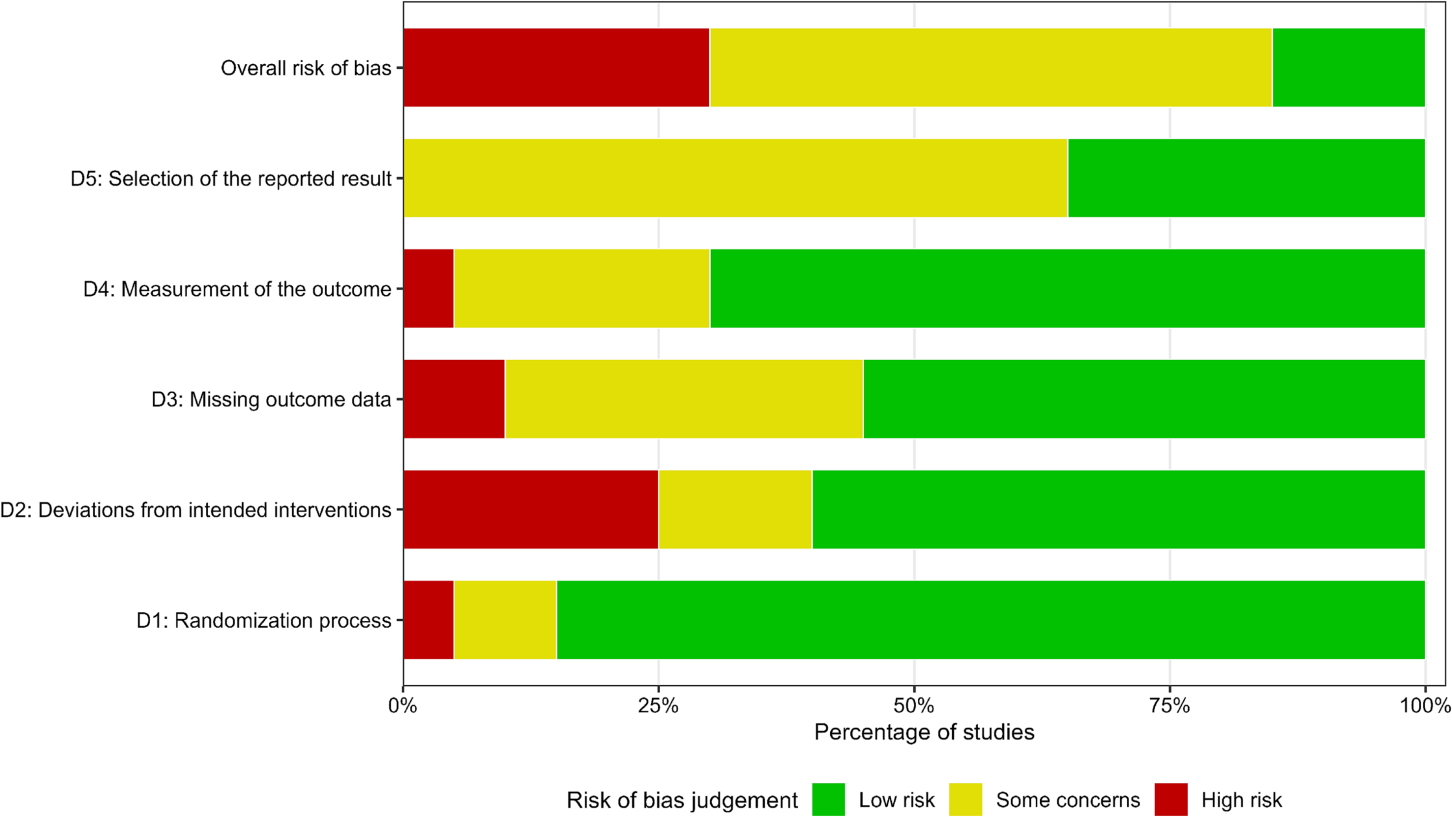
Risk-of-bias summary plot.

Risk-of-bias summaries are shown in Figure 2, with study-specific judgments provided in Supplementary File S3. Using RoB 2, 3 of the 20 studies were judged to be at low overall risk of bias, 11 raised some concerns, and 6 were judged to be at high risk. Most studies were at low risk for the randomization process and measurement of the outcome. The main concerns related to deviations from intended interventions, missing outcome data, and selection of the reported result, particularly because prespecified protocols or analysis plans were not consistently available. Because high-risk judgments were not evenly distributed across outcome indicators and the number of studies was limited, we did not conduct a separate exclusion-based sensitivity analysis after removing all high-risk studies. Overall, the RoB 2 assessment indicated variable methodological quality, which should be considered when interpreting the findings.

### Meta-analysis

The meta-analysis was organized into nine model sections, with physical performance and body composition analyzed separately. BF10 values reported in the Results represent parameter-level Bayes factors against the point null value of zero for individual fixed-effect parameters. They should be read as supplementary descriptors of parameter-level evidence and not as formal comparisons between complete models based on marginal likelihood estimation. Therefore, the Results emphasize posterior estimates, 95% HDIs, heterogeneity estimates, and consistency across related findings. Detailed results for each model are presented in Supplementary File S4. Markov chain convergence was adequate in all models based on the Rhat statistic, with values close to 1.00 across results. Therefore, we did not present convergence results in the main text.

### Null model

**Figure 3:**
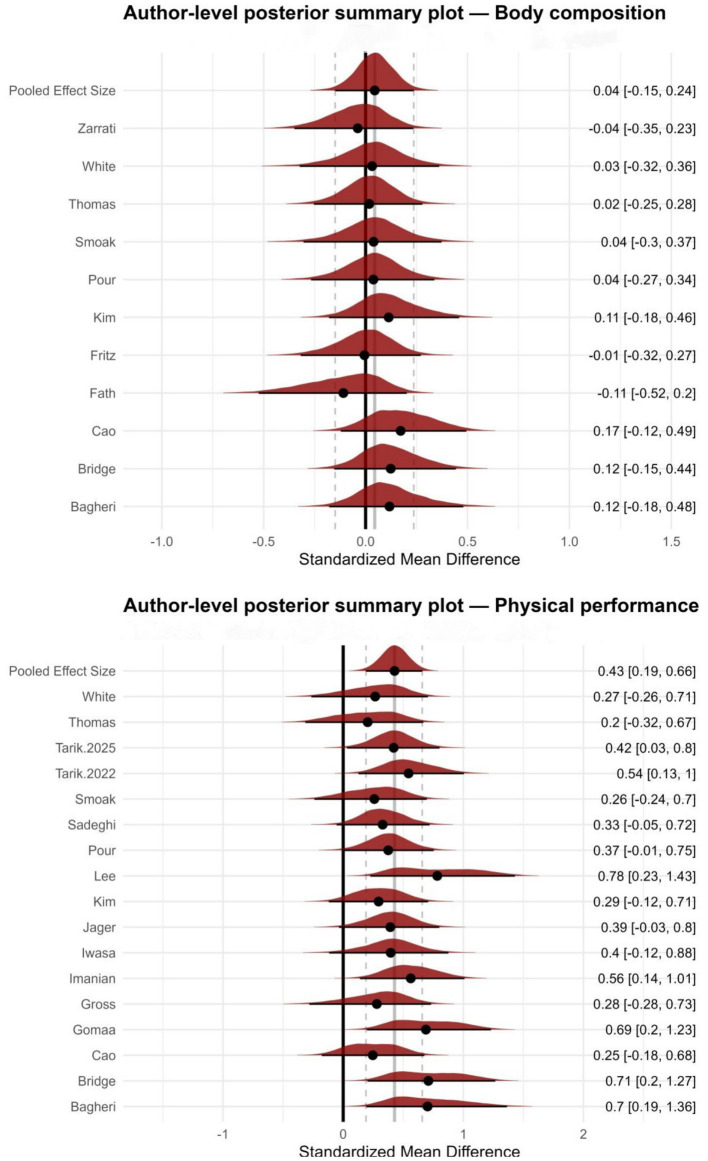
The forest plot in null model.

In the null model, the overall effect size was estimated separately for physical performance and body composition. For physical performance, 17 studies contributed eligible effect sizes to the null model. The posterior summary plot is shown in Figure 3. The posterior estimate suggested a positive domain-level effect for physical performance [Hedges’ *g*: 0.45, 95% HDI: 0.21 to 0.67; parameter-level BF10: 48.98; DI: 5.76; ASI: 0.17], with moderate study-level residual heterogeneity [Tauwithin: 0.27, Taubetween: 0.28]. Because this domain combined several distinct performance indicators, this estimate was interpreted as a broad summary rather than as evidence of uniform improvement across all performance outcomes. For body composition, 11 studies contributed eligible effect sizes to the null model. The posterior summary plot is shown in Figure 3. The Bayesian multilevel meta-analysis suggested that the overall effect was close to null. The posterior estimate for body composition was close to null [Hedges’ *g*: 0.02, 95% HDI: −0.14 to 0.20; parameter-level BF10: 0.21; DI: 4.55; ASI: 0.22], with relatively small study-level residual heterogeneity [Tau_within_: 0.13, Tau_between_: 0.13].

### Outcome indicator model

At the outcome-indicator level, the 95% HDIs excluded zero for muscle strength [Hedges’ *g*: 0.50, 95% HDI: 0.20 to 0.79; parameter-level BF10: 971.97; DI: 3.92; ASI: 0.25] and muscular endurance [Hedges’ *g*: 0.93, 95% HDI: 0.32 to 1.53; parameter-level BF10: 472.68; DI: 1.67; ASI: 0.60], with moderate study-level residual heterogeneity [Tau_within_: 0.12, Tau_between_: 0.41]. Other physical-performance indicators did not show similarly consistent posterior evidence.

**Figure 4:**
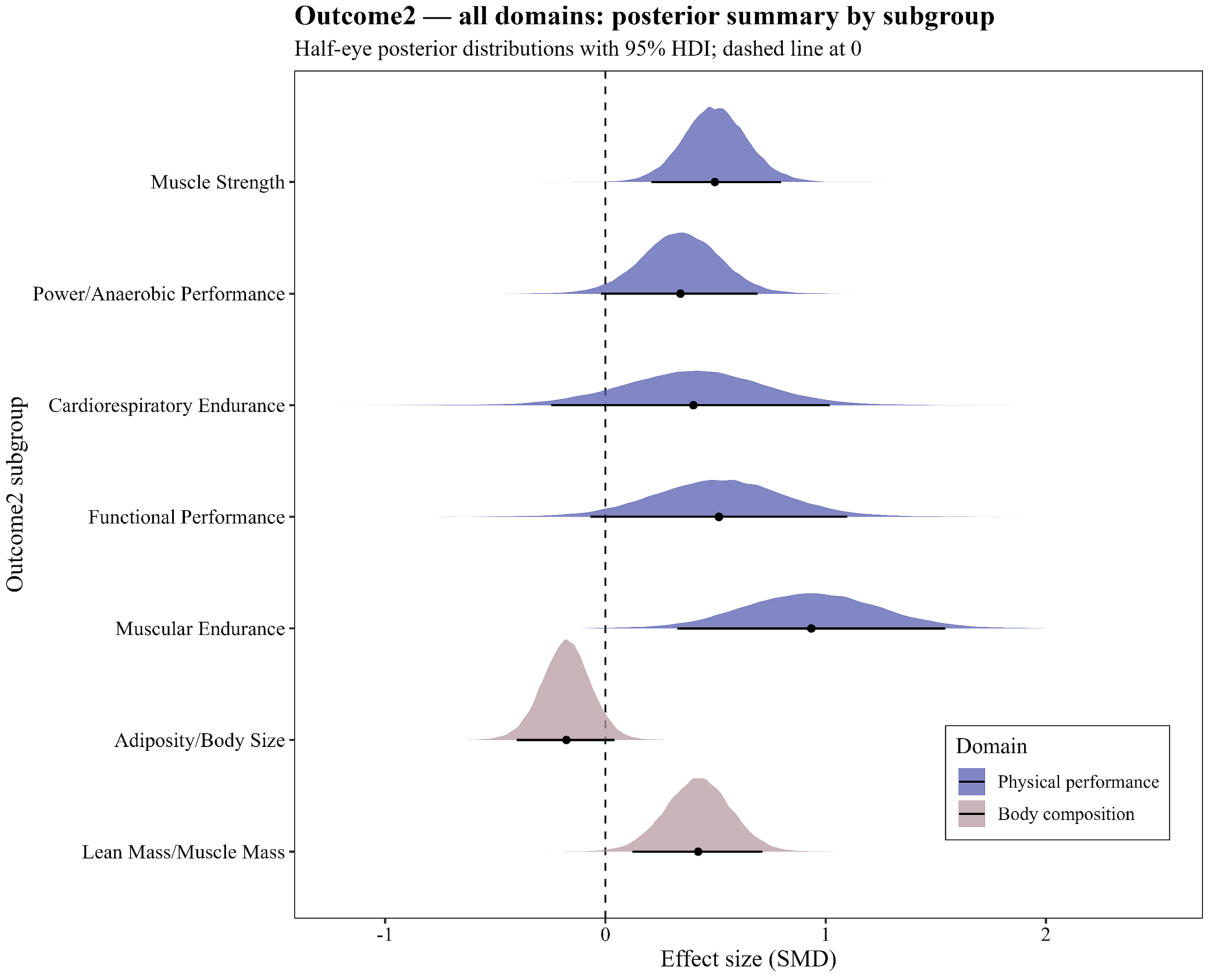
The forest plot in outcome indicator model.

For body composition, the 95% HDI excluded zero only for Lean Mass/Muscle Mass [Hedges’ *g*: 0.42, 95% HDI: 0.13 to 0.72; parameter-level BF10: 278.07; DI: 1.89; ASI: 0.53], with low study-level residual heterogeneity [Tau_within_: 0.11, Tau_between_: 0.10] (Figure 4).

### Control model

For physical performance, protein plus probiotic supplementation showed an effect of [Hedges’ *g*: 0.18, 95% HDI: −0.40 to 0.74; parameter-level BF10: 2.80; DI: 3.00; ASI: 0.33] versus placebo for cardiorespiratory endurance, [Hedges’ *g*: 0.10, 95% HDI: −0.47 to 0.66; parameter-level BF10: 1.71; DI: 1.50; ASI: 0.67] versus placebo for functional performance, [Hedges’ *g*: 0.19, 95% HDI: −0.19 to 0.55; parameter-level BF10: 5.56; DI: 5.57; ASI: 0.18] versus placebo for muscle strength, [Hedges’ *g*: 0.38, 95% HDI: −0.29 to 1.10; parameter-level BF10: 6.13; DI: 1.00; ASI: 1.00] versus placebo for muscular endurance, and [Hedges’ *g*: 0.08, 95% HDI: −0.34 to 0.50; parameter-level BF10: 1.85; DI: 6.00; ASI: 0.17] versus placebo for power/anaerobic performance. Versus protein alone, the corresponding estimates were [Hedges’ *g*: 0.06, 95% HDI: −0.68 to 0.83; parameter-level BF10: 1.24; DI: 1.00; ASI: 1.00] for cardiorespiratory endurance, [Hedges’ *g*: 0.37, 95% HDI: −0.18 to 0.92; parameter-level BF10: 9.93; DI: 2.00; ASI: 0.50] for functional performance, [Hedges’ *g*: 0.26, 95% HDI: −0.18 to 0.66; parameter-level BF10: 7.44; DI: 2.00; ASI: 0.50] for muscle strength, [Hedges’ *g*: 0.70, 95% HDI: 0.17 to 1.20; parameter-level BF10: 172.91; DI: 2.00; ASI: 0.50] for muscular endurance, and [Hedges’ *g*: 0.25, 95% HDI: −0.20 to 0.69; parameter-level BF10: 6.02; DI: 2.50; ASI: 0.40] for power/anaerobic performance, with study-level residual heterogeneity [Tau_within_: 0.08, Tau_between_: 0.67].

**Figure 5:**
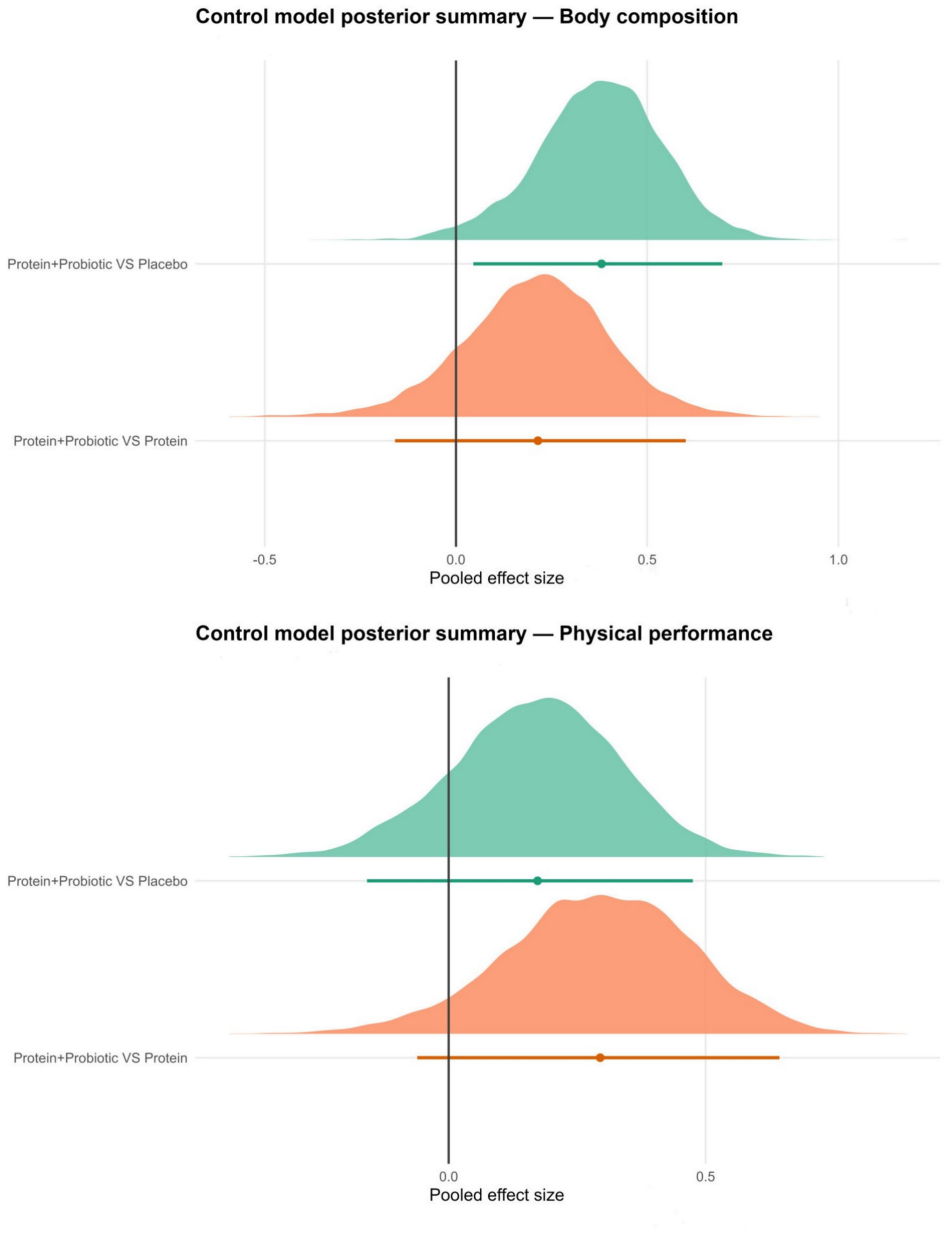
The forest plot in control model.

For body composition, protein plus probiotic supplementation showed an effect of [Hedges’ *g*: 0.31, 95% HDI: −0.04 to 0.64; parameter-level BF10: 23.39; DI: 3.29; ASI: 0.30] versus placebo for adiposity/body size and [Hedges’ *g*: 0.44, 95% HDI: 0.05 to 0.84; parameter-level BF10: 63.00; DI: 1.50; ASI: 0.67] versus placebo for lean mass/muscle mass. Versus protein alone, the corresponding estimates were [Hedges’ *g*: 0.09, 95% HDI: −0.34 to 0.53; parameter-level BF10: 2.08; DI: 3.33; ASI: 0.30] for adiposity/body size and [Hedges’ *g*: 0.34, 95% HDI: −0.08 to 0.76; parameter-level BF10: 15.99; DI: 2.67; ASI: 0.38] for lean mass/muscle mass, with study-level residual heterogeneity [Tau_within_: 0.10, Tau_between_: 0.41] (Figure 5).

### Publication bias

**Figure 6:**
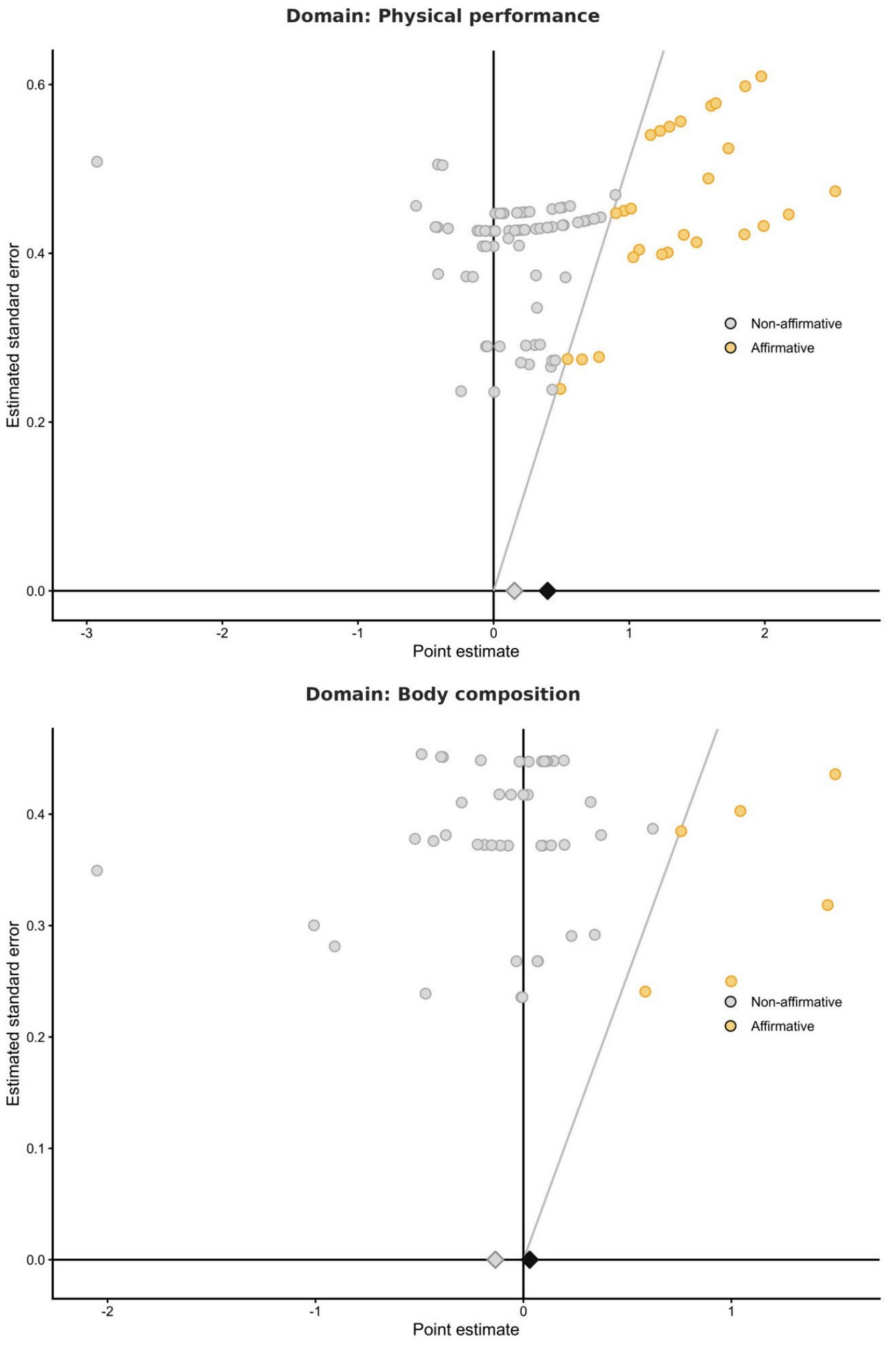
Significance funnel plots for publication bias.

Funnel plots showed some visual asymmetry, but this finding should be interpreted cautiously. The multilevel Egger-type tests did not indicate clear small-study effects for physical performance [*F*(1, 6.03) = 2.87, *p* = 0.141] or body composition [*F*(1, 3.72) = 0.85, *p* = 0.411]. For body composition, studies with non-significant results would need to be reported 1.23 times more frequently than studies with significant results to attenuate the pooled effect toward the null (*s*-value = 1.23). For physical performance, no finite *s*-value was identified within the prespecified range. However, because the number of studies was limited, multiple dependent effect sizes were included within studies, and the outcome indicators were heterogeneous, these publication-bias diagnostics should be regarded as exploratory rather than definitive. The funnel plots are presented in Figure 6, and the detailed publication-bias results are provided in Supplementary File S6.

## Discussion

This review used a Bayesian multilevel meta-analytic framework to synthesize dependent effect sizes contributed by the same trial. This framework is consistent with our previous Bayesian multilevel meta-analysis of protein supplementation in athletes, but the research question was different. The previous review focused on protein supplementation and post-exercise recovery, whereas the present review focused on protein-plus-probiotic co-supplementation and included both physical performance and body composition outcomes. The framework was used to account for the hierarchical structure of the data, whereas the separation of physical performance and body composition, and the further classification into outcome indicators, formed part of the prespecified analytical strategy. This distinction is important because trials of protein-plus-probiotic supplementation often report several correlated endpoints. Retaining these endpoints in a multilevel model reduced the need to discard relevant data while avoiding the assumption that all effect sizes were independent. The indicator-level analyses were then used to improve interpretability and to identify where the most consistent signals were observed. Therefore, the present study extends the previous methodological framework to a different intervention question and adds probiotic-related moderators, including strain composition, specific strain, and delivery form.

### Effects on physical performance

Protein-plus-probiotic supplementation showed a positive domain-level estimate for physical performance, but this estimate should be interpreted as a global summary of exercise-related functional outcomes rather than as evidence that all performance components improved (18, 54). The rationale for retaining a domain-level model was to synthesize the full evidence base while accounting for multiple dependent outcomes within trials. However, muscle strength, muscular endurance, cardiorespiratory endurance, functional performance, and power/anaerobic performance differ in physiological determinants and measurement properties. Therefore, the outcome-indicator model provides the more clinically and practically informative interpretation. In that model, the clearest signals were observed for muscle strength and muscular endurance, whereas evidence for functional performance, cardiorespiratory endurance, and power/anaerobic performance was less consistent (4, 55). The exploratory subgroup findings for population, probiotic strain, protein type, and supplement form should therefore be interpreted as hypothesis-generating (18).

Among athletes, combined supplementation showed possible signals of benefit, but the available evidence did not indicate a consistent population-specific advantage (15, 56). Theoretically, athletes have higher training loads and greater recovery needs, so they are more likely to benefit from the combined effect of protein and probiotics (6, 54). However, in the population stratification results of this study, athletes did not show a particularly clear and consistent advantage pattern. This suggests that the real effect in the athlete population may be jointly affected by training programs, basic diets, supplementation timing, as well as differences in strains and protein types (18, 54). Existing direct studies and reviews also support this cautious interpretation (15, 18, 56). On the one hand, a randomized study of TWK10 combined with pea protein and resistance training reported increased amino acid absorption and partial improvement in physical performance (14). On the other hand, a recent review in the field of sports nutrition on probiotics also pointed out that the impact of probiotics on competitive performance is project – dependent and context dependent, and does not show consistent gains for all athletes and all outcomes (15, 54, 56). Therefore, the positive directions presented by some single-strains and specific protein sources in other models more indicate that combined supplementation may be valuable in specific sports scenarios, rather than being understood as a fully proven general advantage.

Among the elderly, combined protein and probiotic supplementation also shows a certain positive trend in physical performance, but the evidence is still limited. The population model shows that the estimated direction of muscle strength in the elderly is positive, but the interval is wide and the uncertainty is large. This result is physiologically understandable because older adults often show a blunted synthetic response and weaker recovery capacity, and, theoretically, they may be more likely to benefit from nutritional support (57–59). In this regard, classic reviews on anabolic resistance have pointed out that the response of older skeletal muscle to anabolic stimuli such as amino acids and resistance exercise is attenuated (58, 60). Consistently, systematic reviews and meta-analyses in older adults suggest that protein supplementation is more likely to improve appendicular lean mass and grip strength when combined with resistance training, whereas evidence for physical function/performance is less consistent, and benefits tend to be smaller when training is absent (61, 62). Some studies also suggest that probiotics may improve muscle mass and muscle-related function in older adults, but the current results still show substantial heterogeneity or inconsistency (63, 64). Therefore, the findings in older adults should be interpreted as biologically plausible but still weakly supported by direct evidence, and no firm conclusion can be drawn at this stage. For other populations, such as overweight people and samples related to clinical rehabilitation, data are currently limited and the results were sparse and inconsistent (65). Therefore, it is not possible to form a clear judgment on the changes in physical performance in these populations for the time being. The existing results are more suitable to be regarded as exploratory signals rather than mature conclusions.

Overall, the current evidence suggests a selective pattern for physical performance. The most consistent signals were observed for muscle strength and muscular endurance, while the evidence for other performance indicators and non-adult subgroups remained sparse or inconsistent. These findings should be viewed as preliminary because several subgroup estimates were based on small numbers of studies and participants.

### Effects on body composition

The overall estimate for body composition was close to null, suggesting limited evidence for broad changes in body-composition outcomes. At the indicator level, lean mass or muscle mass showed the most consistent positive signal. This pattern is broadly consistent with previous protein-supplementation literature, in which benefits are more often observed for muscle-related indicators than for fat mass, body weight, or broader body-shape outcomes (4, 17, 55). Therefore, any potential body-composition benefit of protein-plus-probiotic supplementation should be interpreted as selective and preliminary.

Among adults, the clearest positive signals in terms of body composition also come from lean body mass or muscle mass. The population model shows that the more definite improvements in adults are mainly in lean body mass or muscle mass, while fat mass and body shape related indicators do not show a stable advantage. The outcome-indicator model showed a similar pattern, with more consistent signals for muscle-related components and weaker evidence for fat reduction or broader body-shape change in the short term. This pattern is highly consistent with the protein supplementation literature (4, 55). Meta-analyses by Morton et al. (4) and Nunes et al. (55) have both shown that increasing protein intake is more likely to bring about a small additional increase in lean body mass or muscle mass but does not guarantee that all body composition indicators will benefit synchronously. The results of the control model also make this interpretation clearer. Compared with the placebo, combined supplementation shows certain positive signals in body composition, but compared with protein alone, its additional advantage is not stable. This suggests that probiotics may provide some additional benefit for body composition, but current evidence is insufficient to show a consistent advantage beyond protein alone. Some protein types and supplementation forms also support the improvement in the direction of lean body mass, but these results should still be treated with caution (4, 55). Overall, what is most worthy of emphasis among adults is not comprehensive improvement of body composition but a certain promotion of lean body mass or muscle mass may be achieved.

Among athletes, the improvement of body composition has not formed a stable pattern. Although athletes are often considered to pay more attention to body composition management, this study does not show that they have a clear and consistent advantage in this field. This may mean that the early effect of combined supplementation on athletes is more likely to be reflected in functional output and training adaptation, rather than being quickly translated into observable body composition changes (15, 16). On the other hand, athletes originally have a higher training level and a relatively better basic diet, so the room for improvement in body composition is relatively small. Existing sports nutrition reviews also point that out the benefits of protein or micro ecology related interventions in athletes often first appear as recovery, training tolerance or specific functional output, rather than bringing stable and significant body composition changes in the short term (15, 16). Therefore, the positive estimates of some protein types, strains or supplementation forms are more suitable as potential clues rather than stable conclusions.

Among the elderly, body composition is also a field worthy of attention but with insufficient evidence. The elderly often face the risk of muscle mass decline and body composition deterioration, so theoretically, they are more suitable for such combined nutritional interventions (58, 62). Previous studies have shown that protein intervention in the elderly is more likely to improve appendicular lean body mass and grip strength when combined with resistance training, while the additional benefits for total lean body mass or more extensive body composition indicators are not always stable (62, 66). At the same time, some meta-analysis on probiotics suggests that in the elderly or older age groups, probiotics may improve muscle mass and muscle function, but have no clear effect on body-shape-related indicators such as BMI (64, 67, 68). This is consistent with the pattern that the body-composition results of the elderly in this study do not form stable support. Therefore, a more reasonable view at present is that the elderly are one of the key objects for future research, but it cannot be determined at this stage that combined protein and probiotic supplementation can stably improve their body composition (62, 68). For other populations, such as overweight people and samples related to clinical rehabilitation, the evidence on body composition is also relatively limited at present. Due to the small sample size and large differences in research design, the existing results are not sufficient to support further extended interpretation.

Overall, the impact of combined protein and probiotic supplementation on body composition appears weaker than that on physical performance. The most relevant practical implication is that this strategy should be viewed as a targeted approach for supporting muscle-related outcomes, especially lean mass or muscle mass, rather than as a broad intervention for fat loss, body-weight reduction, or overall body-shape change. In practice, combined supplementation may be more appropriate when paired with structured training and adequate protein intake, particularly in adults or physically active populations aiming to support strength-related or muscle-related adaptation. However, because effects may vary by probiotic strain, protein source, delivery form, dose, timing, and intervention duration, these practical implications should be applied cautiously until more direct comparative trials are available.

### Strengths, limitations, and future directions

One strength of this review is that it used a more detailed analytical framework. The study did not only report an overall pooled effect but also examined physical performance and body composition separately and explored specific outcome indicators and study characteristics. This allowed a clearer understanding of where potential benefits were more likely to be observed. It also made the interpretation of the findings more specific and practically relevant.

Several limitations should be considered when interpreting the findings. The screening strategy should also be considered when interpreting the completeness of the evidence base. ASReview was used to prioritize records for human screening, and screening was stopped after 200 consecutive irrelevant records. A post-hoc audit of unscreened records did not identify any potentially relevant record. However, because this audit was conducted after the main screening process and was not a formal recall-validation procedure, the possibility of missed eligible records cannot be fully excluded. The search was also limited to English-language publications, and Chinese-language databases were not searched. This may have introduced language bias or database-selection bias if relevant non-English trials were indexed outside the searched databases. The included trials differed in probiotic strain, protein source, delivery form, participant characteristics, and outcome assessment, which may have reduced comparability across studies. In addition, the methodological quality of the primary trials was variable. Only a minority of studies were judged to be at low overall risk of bias under RoB 2, whereas several studies raised some concerns or were judged to be at high risk. The physical-performance domain also combined several conceptually distinct outcomes, which limit the specificity of the domain-level estimate. Some modeling choices may also affect interpretation. The Bayesian analyses used weakly informative priors, and no formal prior-sensitivity analysis was conducted. Therefore, the robustness of the posterior estimates and Bayes factors to alternative prior specifications could not be directly evaluated. For this reason, Bayes factors were treated as supplementary descriptors rather than as the primary basis for inference. In addition, the multilevel models retained dependent effect sizes through study-level and within-study random effects, but the exact within-study covariance structure was not available from the primary reports. This may have affected the precision of some estimates. Publication-bias diagnostics were further constrained by the limited number of studies, dependent effect sizes within studies, and outcome heterogeneity. Therefore, although the present review identified some promising signals, the findings should be interpreted with appropriate caution, particularly for subgroup-, strain-, and indicator-specific results.

Future trials should use more standardized and transparent reporting. Studies should clearly report dose, timing, probiotic strain, protein source, and intervention context. Better reporting in these areas will help identify which combined strategies are most effective and under what conditions they work best.

## Conclusion

Overall, the available evidence suggests possible selective benefits of protein-plus-probiotic co-supplementation, mainly for physical performance and for muscle strength, muscular endurance, and lean mass or muscle mass at the indicator level. Evidence for broad body-composition changes remains limited. Given the small number of studies in several subgroups, variable risk of bias, outcome heterogeneity, and uncertainty in some estimates, these findings should be considered preliminary. Further well-designed trials are needed before firm conclusions can be made about specific strains, protein sources, delivery forms, or target populations.

## Data Availability

The original contributions presented in the study are included in the article/Supplementary material, further inquiries can be directed to the corresponding author.
