# Supplementary material for: Effects of combined protein and probiotic supplementation on physical performance and body composition: a Bayesian multilevel meta-analysis of randomized controlled trials
Source: Front Nutr. 2026 Jun 17;13:1865035. doi: 10.3389/fnut.2026.1865035 (PMC13319104; doi:10.3389/fnut.2026.1865035)
Supplement: Supplementary file 1 [file Table_1.DOCX]

**Supplementary File S1: Search Strategy**

| Pubmed | |
| --- | --- |
|  | ("Probiotics"[Mesh] OR probiotics[tiab] OR probiotic[tiab]  OR "Lactic Acid Bacteria"[Mesh] OR lactic acid bacteri*[tiab]  OR "Lactobacillus"[Mesh] OR lactobacillus[tiab]  OR "Bifidobacterium"[Mesh] OR bifidobacterium[tiab]  OR "Yeast"[Mesh] OR yeast*[tiab]  OR "Yogurt"[Mesh] OR yogurt[tiab] OR yoghurt[tiab] OR "Greek yogurt"[tiab]  OR kefir[tiab] OR "Fermented Milk"[tiab] OR "Fermented Dairy"[tiab]  OR "Soy Yogurt"[tiab] OR "Almond Yogurt"[tiab])  AND (protein[Mesh] OR protein[tiab] OR proteins[tiab]  OR "Dietary Proteins"[Mesh] OR "Dietary Protein"[tiab]  OR whey[tiab] OR casein[tiab] OR leucine[Mesh] OR leucine[tiab]  OR "Branched-Chain Amino Acids"[Mesh] OR "branched-chain amino acid"[tiab]  OR amino acid*[tiab] OR supplement[tiab] OR supplements[tiab])  AND ("Body Composition"[Mesh] OR "body composition"[tiab]  OR "Lean Mass"[tiab] OR "Fat-Free Mass"[tiab] OR "Fat Free Mass"[tiab]  OR "Fat Mass"[tiab] OR "Muscle Mass"[tiab] OR "Skeletal Muscle"[tiab]  OR "Muscle Strength"[Mesh] OR "muscle strength"[tiab]  OR VO2max[tiab] OR "VO2 max"[tiab]  OR "Aerobic Capacity"[tiab] OR jump*[tiab] OR sprint*[tiab]  OR performance[tiab] OR "exercise performance"[tiab] OR "physical performance"[tiab]) |

| Web of Science | |
| --- | --- |
|  | TS=(  probiotics OR probiotic OR synbiotic* OR "lactic acid bacteria"  OR lactobacillus OR lacticaseibacillus OR lactiplantibacillus OR limosilactobacillus OR levilactobacillus OR ligilactobacillus  OR bifidobacterium OR "saccharomyces boulardii" OR "streptococcus thermophilus" OR "lactococcus lactis" OR "enterococcus faecium"  OR "bacillus coagulans" OR "bacillus clausii" OR "clostridium butyricum" OR "E. coli nissle" OR "nissle 1917"  OR LGG OR NCFM OR "299v" OR "BB-12" OR BB536 OR HN019 OR B420 OR "DSM 17938" OR Shirota OR "CNCM I-745" OR "UBBC-07" OR "MIYAIRI 588" OR "CBM 588" OR BC30 OR "GBI-30"  OR yogurt OR yoghurt OR "Greek yogurt" OR kefir OR "fermented milk" OR "fermented dairy" OR "fermented yogurt" OR "soy yogurt" OR "plant-based yogurt" OR "almond yogurt"  )  AND TS=(  protein OR proteins OR "protein supplement*" OR "protein supplementation" OR "dietary protein*"  OR "whey protein*" OR casein OR "milk protein*" OR "plant protein*" OR "soy protein*" OR "pea protein*" OR "rice protein*" OR "wheat protein*"  OR EAA OR "essential amino acid*" OR BCAA OR leucine OR isoleucine OR valine  )  AND TS=(  "body composition" OR "lean mass" OR "fat-free mass" OR FFM OR "skeletal muscle mass"  OR "fat mass" OR "body fat percent*" OR BMI OR "waist circumference" OR WC OR "waist-hip ratio" OR WHR OR skinfold*  OR DXA OR DEXA OR BIA OR "bioelectrical impedance"  OR "bone mineral density" OR BMD OR "bone mineral content" OR BMC  OR "muscle strength" OR handgrip OR "1RM" OR isometric OR isokinetic OR power OR "rate of force development" OR RFD OR "peak torque"  OR "vertical jump" OR CMJ OR "countermovement jump" OR SJ OR DJ OR sprint* OR "repeated sprint" OR RSA OR agility OR "T-test" OR "Illinois agility"  OR VO2max OR "VO2 max" OR "time to exhaustion" OR "time trial"  OR "running economy" OR "cycling economy" OR "ventilatory threshold" OR "lactate threshold"  OR Wingate OR "peak power output" OR PPO OR "mean power output" OR MPO  ) |

| Scopus | |
| --- | --- |
|  | TITLE-ABS-KEY(  probiotics OR probiotic OR synbiotic* OR "lactic acid bacteria"  OR lactobacillus OR lacticaseibacillus OR lactiplantibacillus OR limosilactobacillus OR levilactobacillus OR ligilactobacillus  OR bifidobacterium OR "saccharomyces boulardii" OR "streptococcus thermophilus" OR "lactococcus lactis" OR "enterococcus faecium"  OR "bacillus coagulans" OR "bacillus clausii" OR "clostridium butyricum" OR "E. coli nissle" OR "nissle 1917"  OR LGG OR NCFM OR "299v" OR "BB-12" OR BB536 OR HN019 OR B420 OR "DSM 17938" OR Shirota OR "CNCM I-745" OR "UBBC-07" OR "MIYAIRI 588" OR "CBM 588" OR BC30 OR "GBI-30"  OR yogurt OR yoghurt OR "Greek yogurt" OR kefir OR "fermented milk" OR "fermented dairy" OR "fermented yogurt" OR "soy yogurt" OR "plant-based yogurt" OR "almond yogurt"  )  AND TITLE-ABS-KEY(  protein OR proteins OR "protein supplement*" OR "protein supplementation" OR "dietary protein*"  OR "whey protein*" OR casein OR "milk protein*" OR "plant protein*" OR "soy protein*" OR "pea protein*" OR "rice protein*" OR "wheat protein*"  OR EAA OR "essential amino acid*" OR BCAA OR leucine OR isoleucine OR valine  )  AND TITLE-ABS-KEY(  "body composition" OR "lean mass" OR "fat-free mass" OR FFM OR "skeletal muscle mass"  OR "fat mass" OR "body fat percent*" OR BMI OR "waist circumference" OR WC OR "waist-hip ratio" OR WHR OR skinfold*  OR DXA OR DEXA OR BIA OR "bioelectrical impedance"  OR "bone mineral density" OR BMD OR "bone mineral content" OR BMC  OR "muscle strength" OR handgrip OR "1RM" OR isometric OR isokinetic OR power OR "rate of force development" OR RFD OR "peak torque"  OR "vertical jump" OR CMJ OR "countermovement jump" OR SJ OR DJ OR sprint* OR "repeated sprint" OR RSA OR agility OR "T-test" OR "Illinois agility"  OR VO2max OR "VO2 max" OR "time to exhaustion" OR "time trial"  OR "running economy" OR "cycling economy" OR "ventilatory threshold" OR "lactate threshold"  OR Wingate OR "peak power output" OR PPO OR "mean power output" OR MPO  ) |

| EBSCO (SPORTDiscus, CINAHL Complete) | |
| --- | --- |
|  | TI,AB(  probiotic* OR synbiotic* OR "lactic acid bacteria"  OR lactobacillus OR lacticaseibacillus OR lactiplantibacillus OR limosilactobacillus OR levilactobacillus OR ligilactobacillus  OR bifidobacterium OR "saccharomyces boulardii" OR "streptococcus thermophilus" OR "lactococcus lactis" OR "enterococcus faecium"  OR "bacillus coagulans" OR "bacillus clausii" OR "clostridium butyricum" OR "E. coli nissle" OR "nissle 1917"  OR LGG OR NCFM OR 299v OR "BB-12" OR BB536 OR HN019 OR B420 OR "DSM 17938" OR Shirota OR "CNCM I-745" OR "UBBC-07" OR "MIYAIRI 588" OR "CBM 588" OR BC30 OR "GBI-30"  OR yogurt OR yoghurt OR "Greek yogurt" OR kefir OR "fermented milk" OR "fermented dairy" OR "fermented yogurt" OR "soy yogurt" OR "plant-based yogurt" OR "almond yogurt"  )  AND TI,AB(  protein OR proteins OR ("protein" W1 supplement*) OR "protein supplementation" OR ("dietary" W1 protein*)  OR ("whey" W1 protein*) OR casein OR ("milk" W1 protein*)  OR ("plant" W1 protein*) OR ("soy" W1 protein*) OR ("pea" W1 protein*) OR ("rice" W1 protein*) OR ("wheat" W1 protein*)  OR EAA OR ("essential" W1 "amino" W1 acid*) OR BCAA OR leucine OR isoleucine OR valine  )  AND TI,AB(  "body composition" OR "lean mass" OR "fat-free mass" OR FFM OR "skeletal muscle mass"  OR "fat mass" OR "body fat percent*" OR BMI OR "waist circumference" OR WC OR "waist-hip ratio" OR WHR OR skinfold*  OR DXA OR DEXA OR BIA OR "bioelectrical impedance"  OR "bone mineral density" OR BMD OR "bone mineral content" OR BMC  OR "muscle strength" OR handgrip OR "1RM" OR isometric OR isokinetic OR power OR "rate of force development" OR RFD OR "peak torque"  OR "vertical jump" OR CMJ OR "countermovement jump" OR SJ OR DJ OR sprint* OR "repeated sprint" OR RSA OR agility OR "T-test" OR "Illinois agility"  OR VO2max OR "VO2 max" OR "time to exhaustion" OR "time trial"  OR "running economy" OR "cycling economy" OR "ventilatory threshold" OR "lactate threshold"  OR Wingate OR "peak power output" OR PPO OR "mean power output" OR MPO  ) |

| Cochrane Library | | |
| --- | --- | --- |
|  | | (  probiotics OR probiotic OR synbiotic* OR "lactic acid bacteria" OR lactobacillus OR lacticaseibacillus OR lactiplantibacillus  OR limosilactobacillus OR levilactobacillus OR ligilactobacillus  OR bifidobacterium OR "saccharomyces boulardii" OR "streptococcus thermophilus" OR "lactococcus lactis" OR "enterococcus faecium"  OR "bacillus coagulans" OR "bacillus clausii" OR "clostridium butyricum" OR "E. coli nissle" OR "nissle 1917"  OR LGG OR NCFM OR 299v OR "BB-12" OR BB536 OR HN019 OR B420 OR "DSM 17938" OR Shirota OR "CNCM I-745" OR "UBBC-07" OR "MIYAIRI 588" OR "CBM 588" OR BC30 OR "GBI-30"  OR yogurt OR yoghurt OR "Greek yogurt" OR kefir OR "fermented milk" OR "fermented dairy" OR "fermented yogurt" OR "soy yogurt" OR "plant-based yogurt" OR "almond yogurt"  )  AND  (  protein OR proteins OR (protein NEXT supplement*) OR "protein supplementation" OR (dietary NEXT protein*)  OR (whey NEXT protein*) OR casein OR (milk NEXT protein*) OR (plant NEXT protein*) OR (soy NEXT protein*) OR (pea NEXT protein*) OR (rice NEXT protein*) OR (wheat NEXT protein*)  OR EAA OR (essential NEXT amino NEXT acid*) OR BCAA OR leucine OR isoleucine OR valine  )  AND  (  "body composition" OR "lean mass" OR "fat-free mass" OR FFM OR "skeletal muscle mass"  OR "fat mass" OR (body NEXT fat NEXT percent*) OR BMI OR "waist circumference" OR WC OR "waist-hip ratio" OR WHR OR skinfold*  OR DXA OR DEXA OR BIA OR "bioelectrical impedance"  OR "bone mineral density" OR BMD OR "bone mineral content" OR BMC  OR "muscle strength" OR handgrip OR 1RM OR isometric OR isokinetic OR power OR "rate of force development" OR RFD OR "peak torque"  OR "vertical jump" OR CMJ OR "countermovement jump" OR SJ OR DJ OR sprint* OR "repeated sprint" OR RSA OR agility OR "T-test" OR "Illinois agility"  OR VO2max OR "VO2 max" OR "time to exhaustion" OR "time-to-exhaustion" OR "time trial"  OR "running economy" OR "cycling economy" OR "ventilatory threshold" OR "lactate threshold"  OR Wingate OR "peak power output" OR PPO OR "mean power output" OR MPO) |
| Embase | | |
|  | (  'probiotic agent'/exp  OR 'lactobacillus'/exp  OR 'bifidobacterium'/exp  OR 'yeast'/exp  OR 'yogurt'/exp  OR 'kefir'/exp  OR 'fermented milk'/exp  OR 'Probiotics':ti,ab OR probiotics:ti,ab OR probiotic:ti,ab  OR 'Lactic Acid Bacteria':ti,ab OR 'lactic acid bacteri*':ti,ab  OR 'Lactobacillus':ti,ab OR lactobacillus:ti,ab  OR 'Bifidobacterium':ti,ab OR bifidobacterium:ti,ab  OR 'Yeast':ti,ab OR yeast*:ti,ab  OR 'Yogurt':ti,ab OR yogurt:ti,ab OR yoghurt:ti,ab OR 'Greek yogurt':ti,ab  OR kefir:ti,ab OR 'Fermented Milk':ti,ab OR 'Fermented Dairy':ti,ab  OR 'Soy Yogurt':ti,ab OR 'Almond Yogurt':ti,ab  )  AND  (  'protein'/exp  OR 'dietary protein'/exp  OR 'whey protein'/exp  OR 'casein'/exp  OR 'leucine'/exp  OR 'branched chain amino acid'/exp  OR 'amino acid'/exp  OR 'dietary supplement'/exp  OR protein:ti,ab OR proteins:ti,ab  OR 'Dietary Proteins':ti,ab OR 'Dietary Protein':ti,ab  OR whey:ti,ab OR casein:ti,ab OR leucine:ti,ab  OR 'Branched-Chain Amino Acids':ti,ab OR 'branched-chain amino acid':ti,ab  OR 'amino acid*':ti,ab OR supplement:ti,ab OR supplements:ti,ab  )  AND  (  'body composition'/exp  OR 'skeletal muscle'/exp  OR 'muscle strength'/exp  OR 'physical performance'/exp  OR 'athletic performance'/exp  OR 'Body Composition':ti,ab OR 'body composition':ti,ab  OR 'Lean Mass':ti,ab OR 'Fat-Free Mass':ti,ab OR 'Fat Free Mass':ti,ab  OR 'Fat Mass':ti,ab OR 'Muscle Mass':ti,ab OR 'Skeletal Muscle':ti,ab  OR 'Muscle Strength':ti,ab OR 'muscle strength':ti,ab  OR VO2max:ti,ab OR 'VO2 max':ti,ab  OR 'Aerobic Capacity':ti,ab OR jump*:ti,ab OR sprint*:ti,ab  OR performance:ti,ab OR 'exercise performance':ti,ab OR 'physical performance':ti,ab  ) | |
