# Supplementary material for: Effects of combined protein and probiotic supplementation on physical performance and body composition: a Bayesian multilevel meta-analysis of randomized controlled trials
Source: Front Nutr. 2026 Jun 17;13:1865035. doi: 10.3389/fnut.2026.1865035 (PMC13319104; doi:10.3389/fnut.2026.1865035)
Supplement: Supplementary file 3 [file Table_3.DOCX]

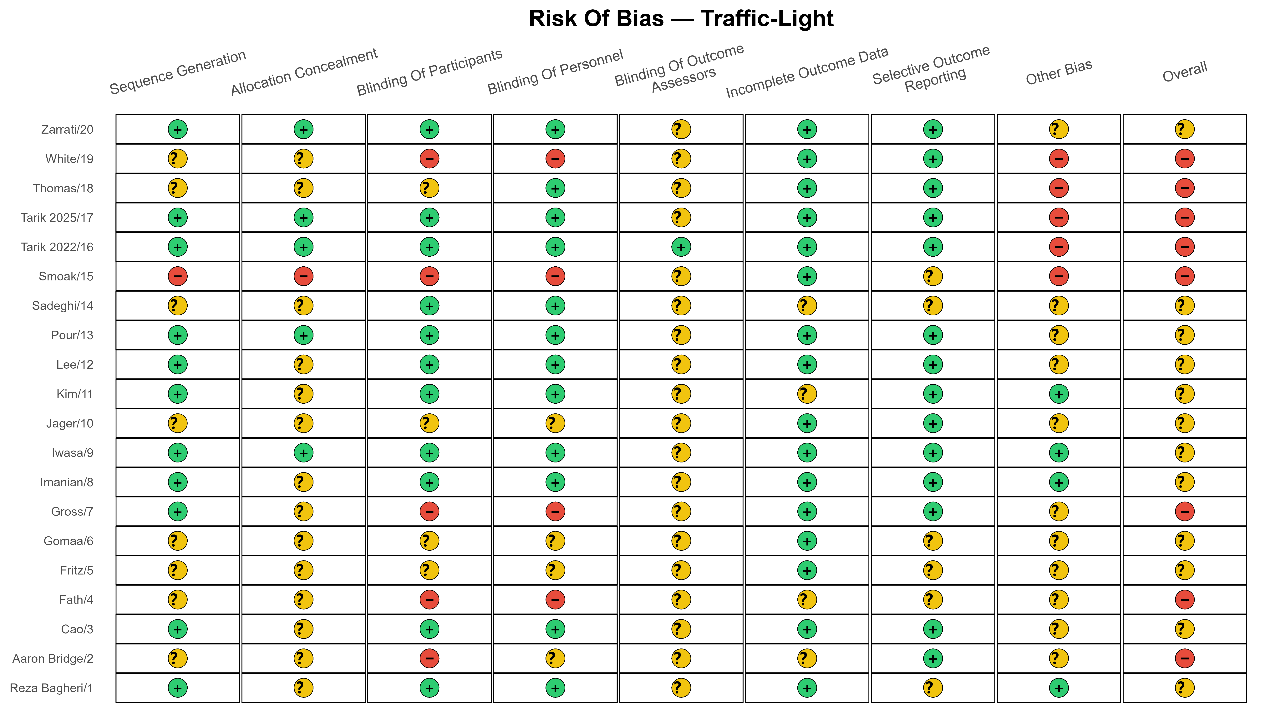


**Fig. S1** Risk of bias with study specific plots


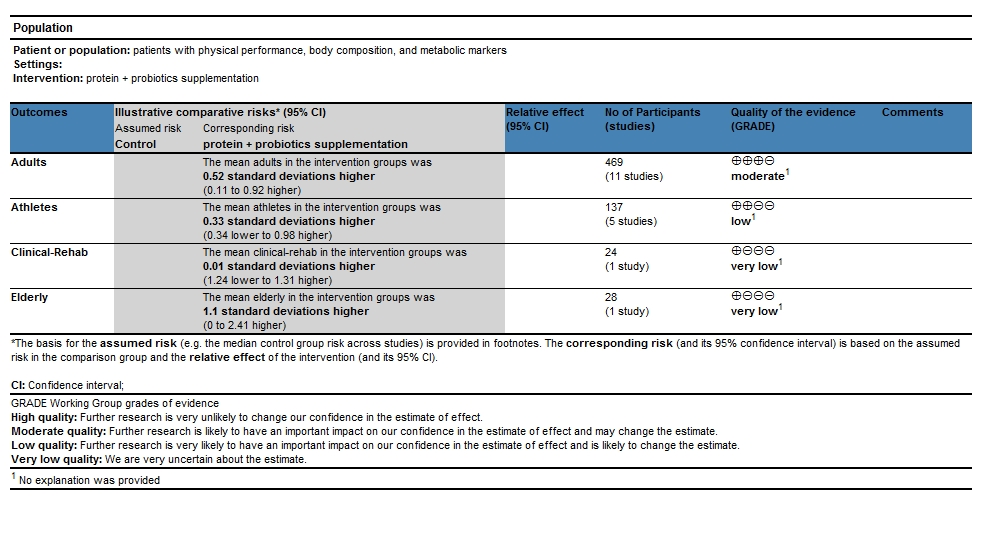


**Fig. S2** Quality Grade of Population Model


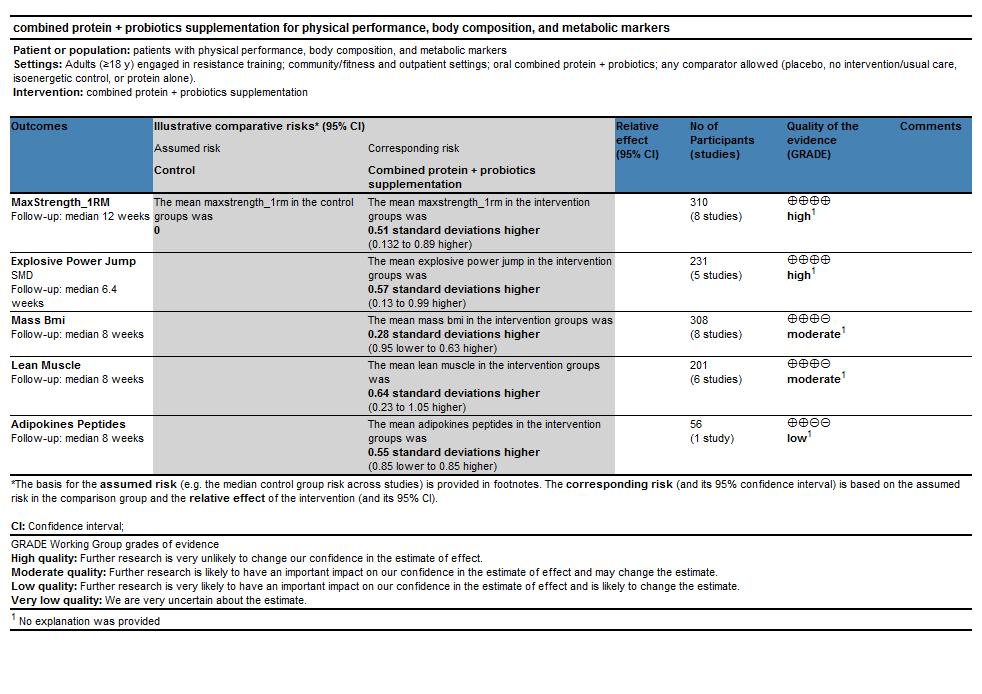


**Fig. S3** Quality Grade of Outcome Domain Model


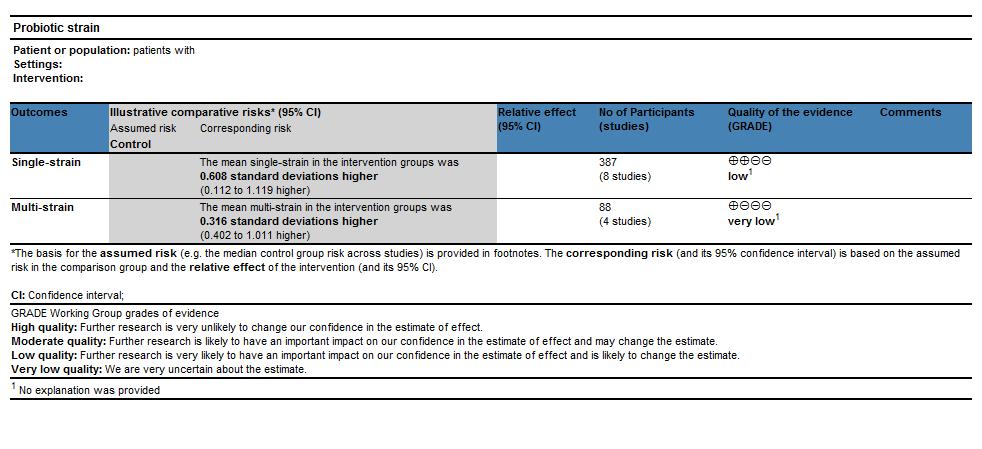


**Fig. S4** Quality Grade of Probiotic Preparation Type Model


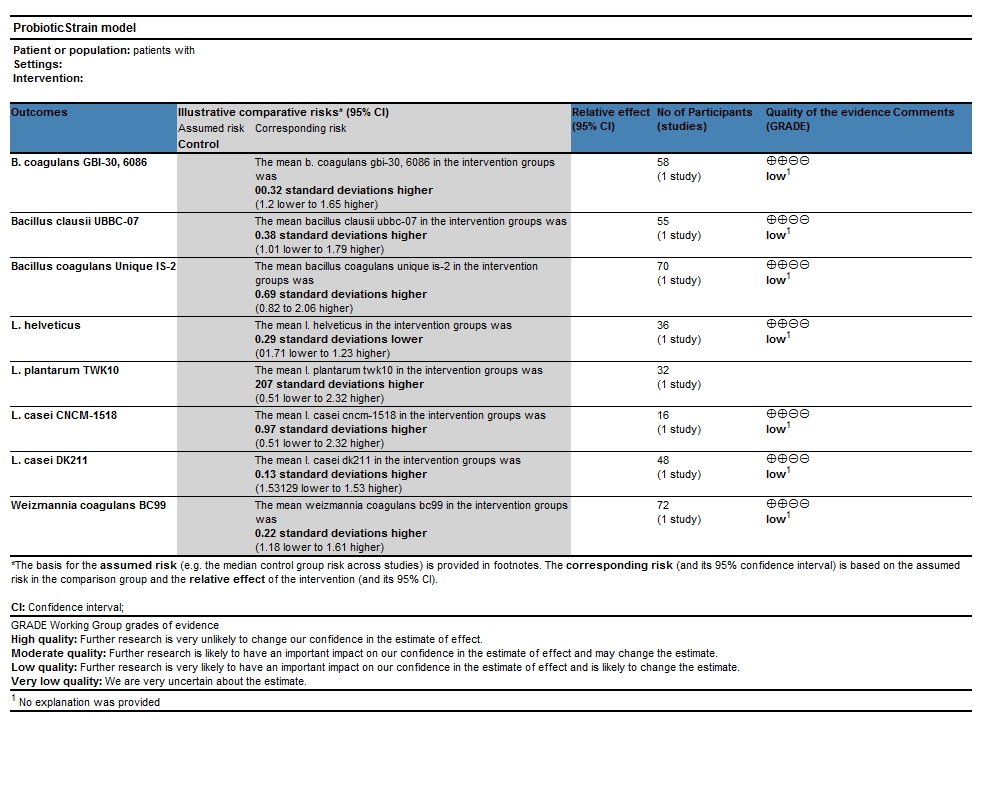


**Fig. S5** Quality Grade of Probiotic Single-strain Species Model


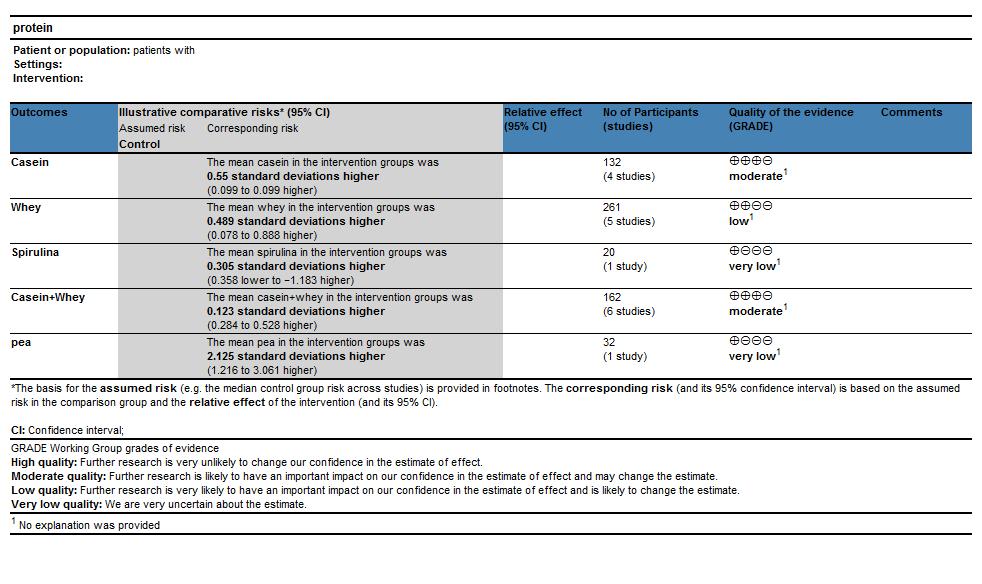


**Fig. S6** Quality Grade of Protein Category Model


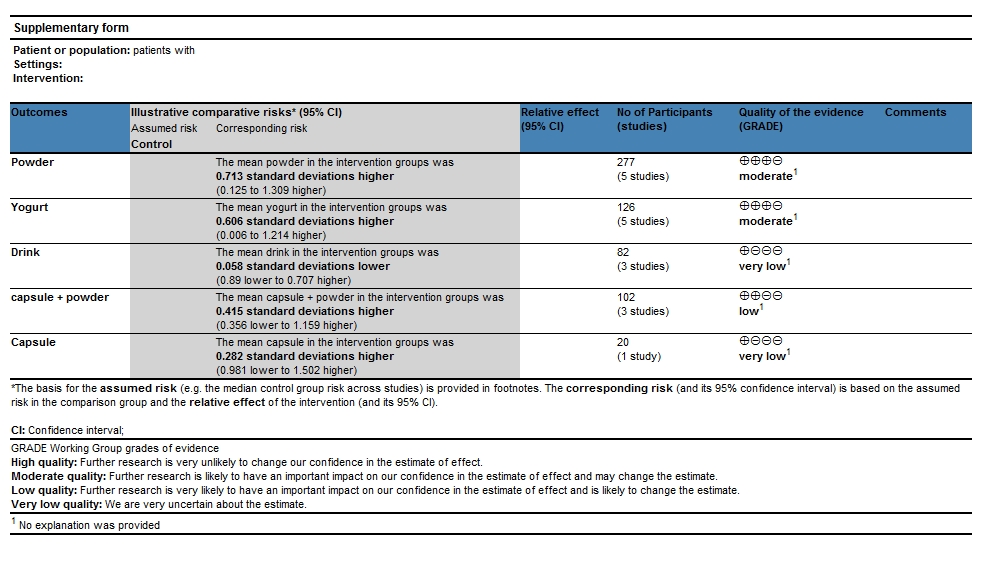


**Fig. S7** Quality Grade of Supplement Form Model


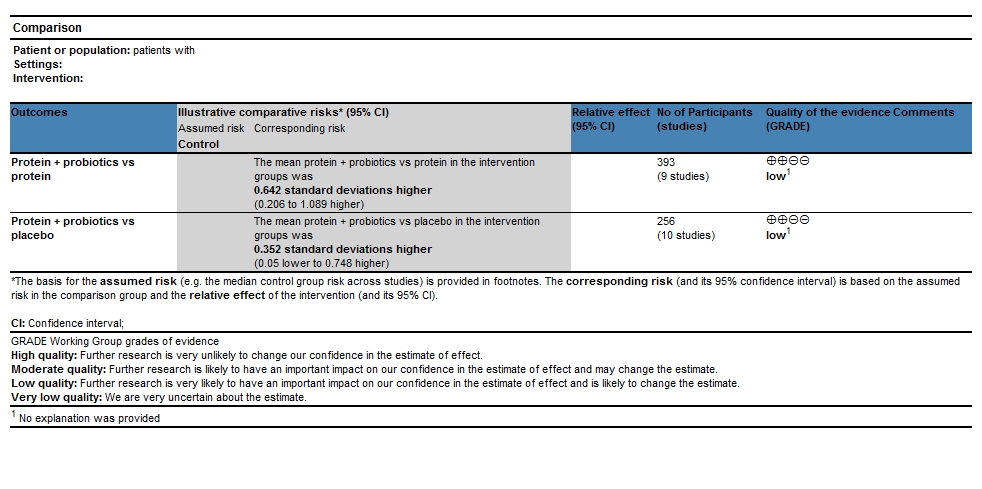


**Fig. S8** Quality Grade of Control Type Model


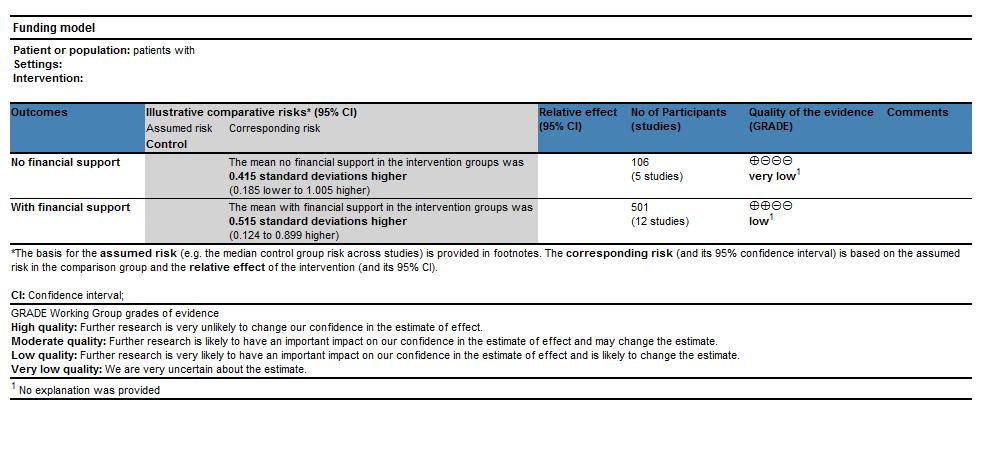


**Fig. S9** Quality Grade of Funding Source Model
