# Supplementary material for: Effects of combined protein and probiotic supplementation on physical performance and body composition: a Bayesian multilevel meta-analysis of randomized controlled trials
Source: Front Nutr. 2026 Jun 17;13:1865035. doi: 10.3389/fnut.2026.1865035 (PMC13319104; doi:10.3389/fnut.2026.1865035)

**Supplementary File S5: Forest-style posterior summaries and Bayes factor plots for exploratory models, including Population, Supplementary Form, Probiotic Strain/Type, Protein Type, and Funding across Physical Performance And Body Composition Outcomes.**

Fig. S1 Posterior summary plot for the Population Model in physical performance.


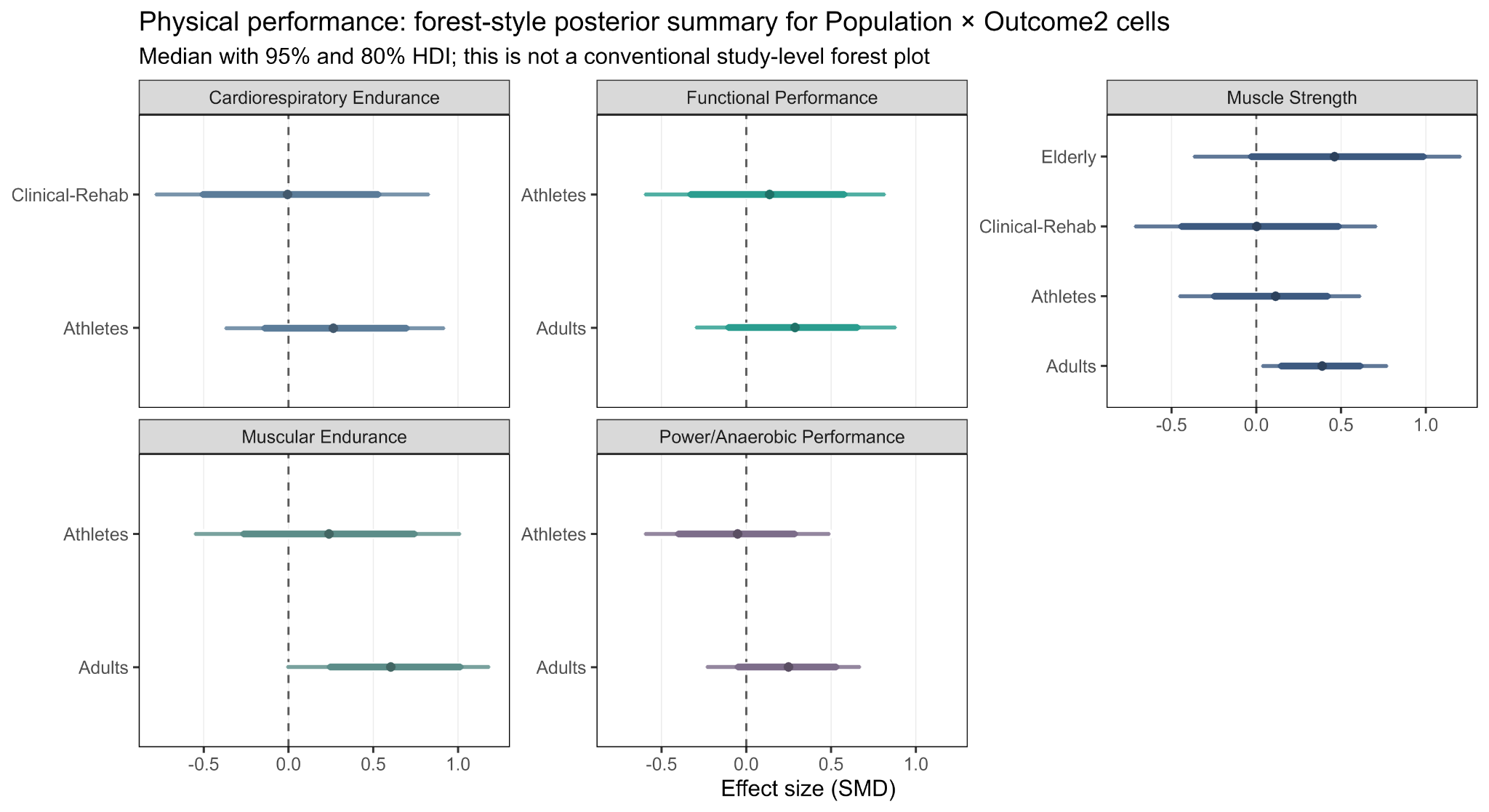


Fig. S2 Posterior summary plot for the Population Model in body composition.


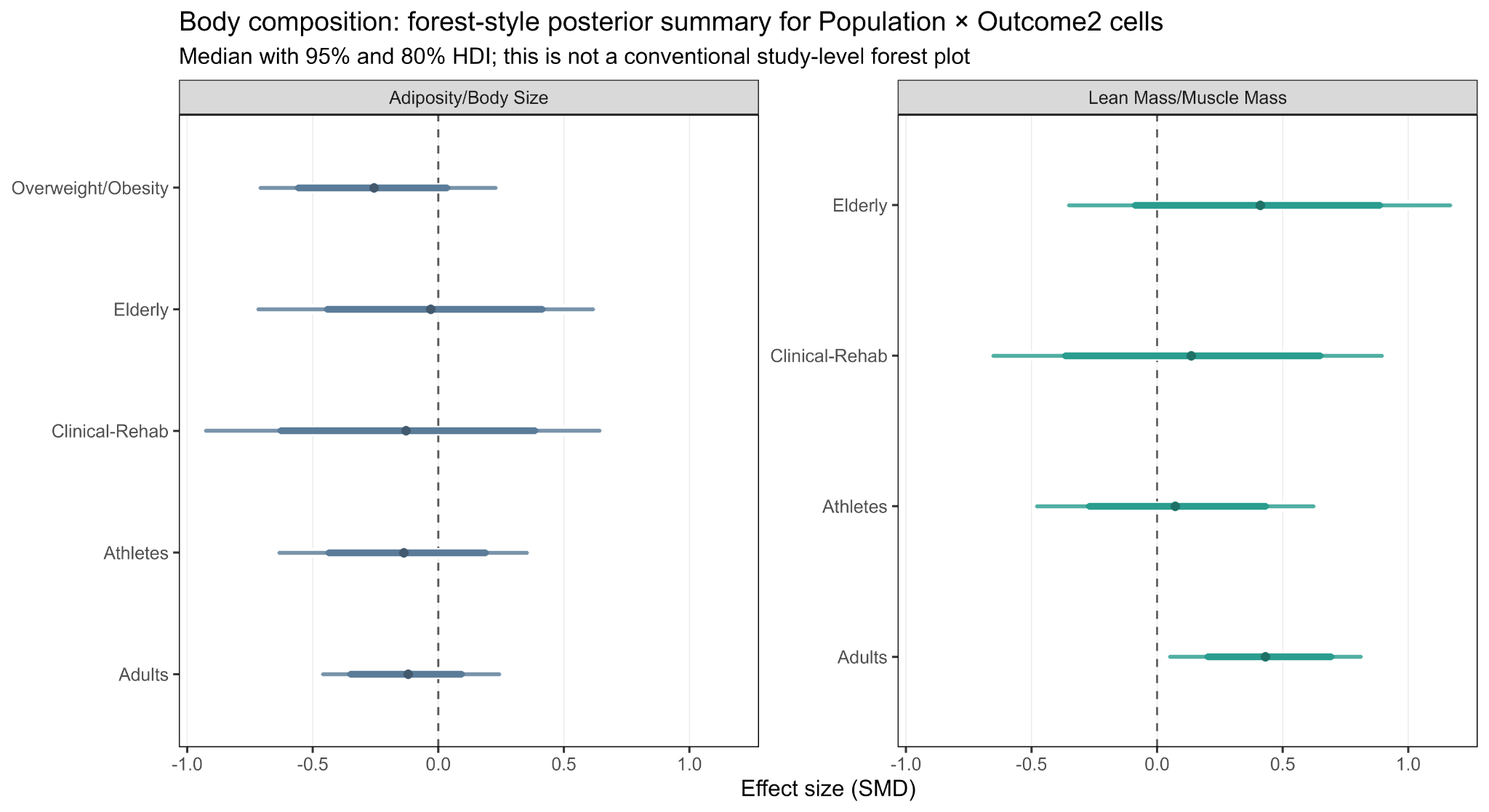


Fig. S3 Bayes factor plot of the Population Model.


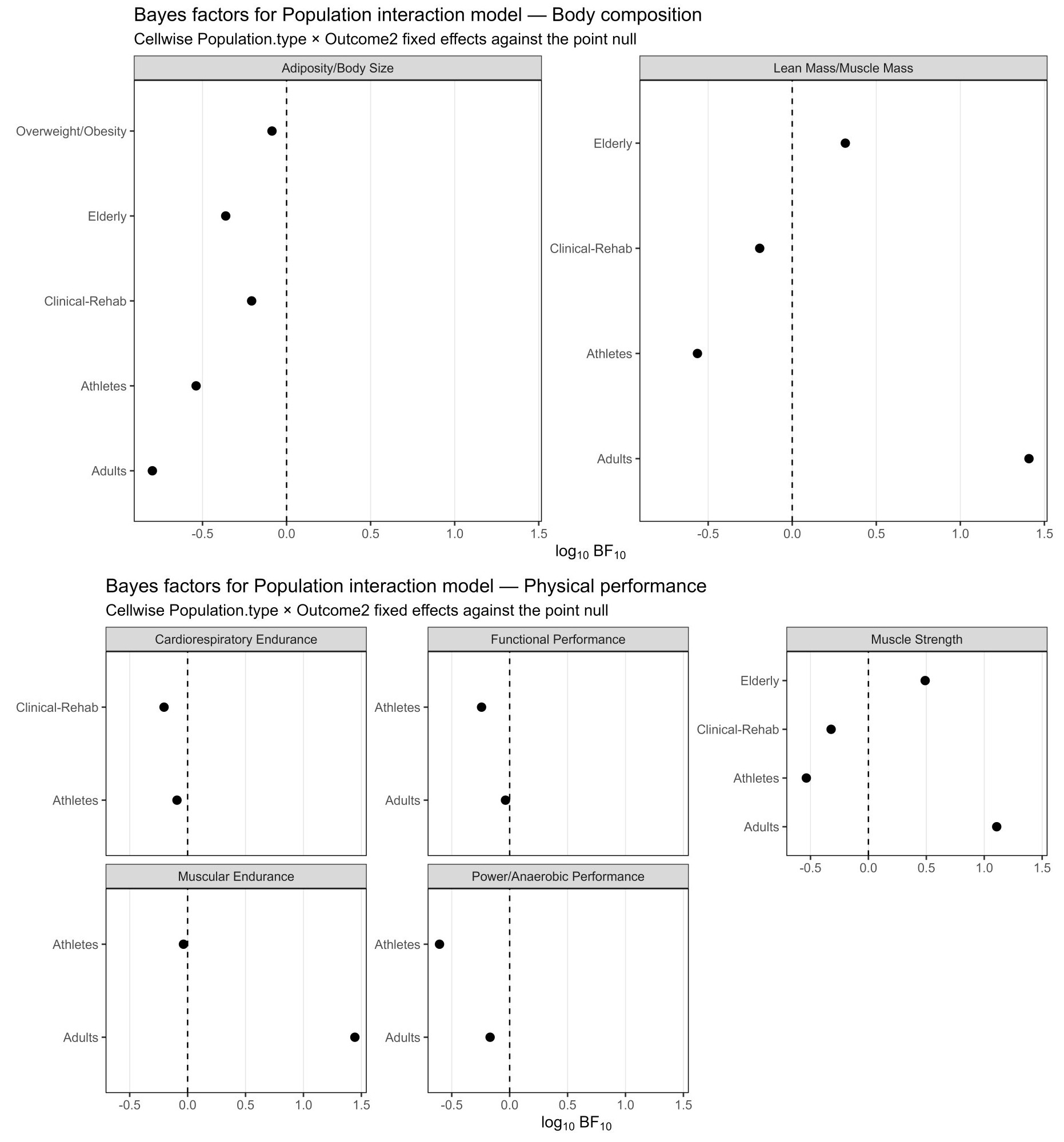


Fig. S4 Posterior summary plot for the Supplement Form Model across physical performance and body composition outcomes.


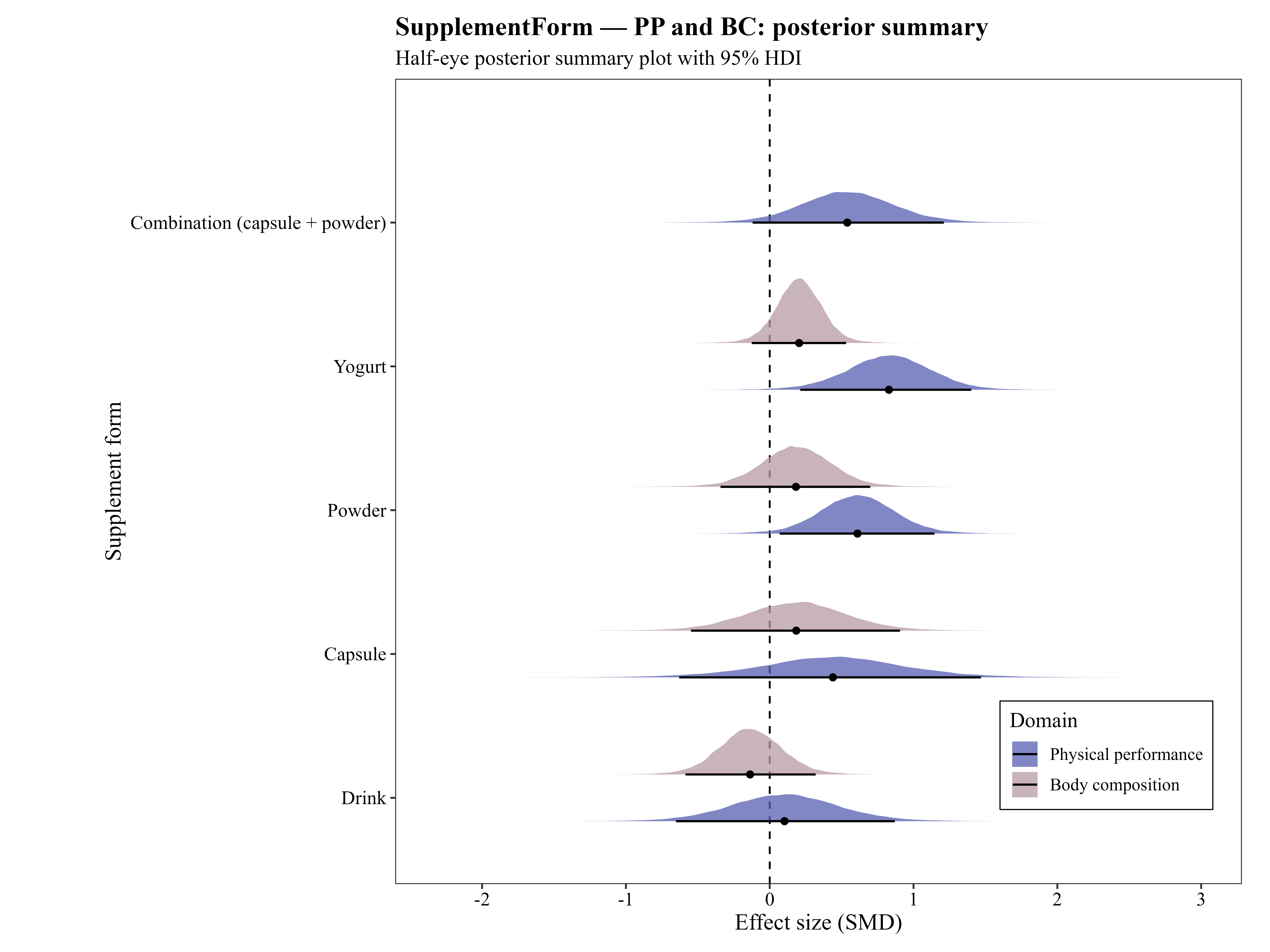


Fig. S5 Bayes factor plot of the Supplementary Form Model.


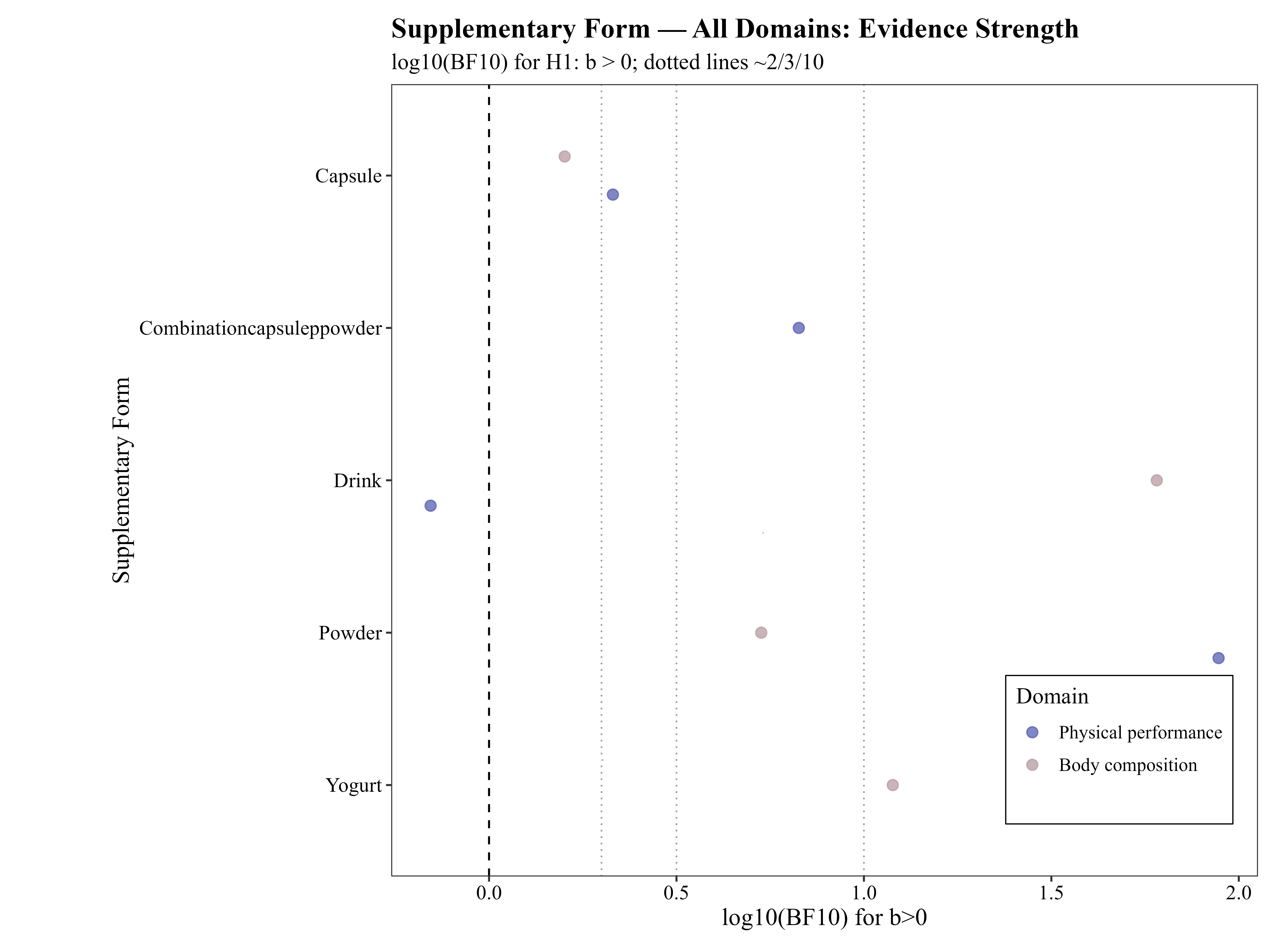


Fig. S6 Posterior summary plot for the Probiotic Preparation Model across physical performance and body composition outcomes.


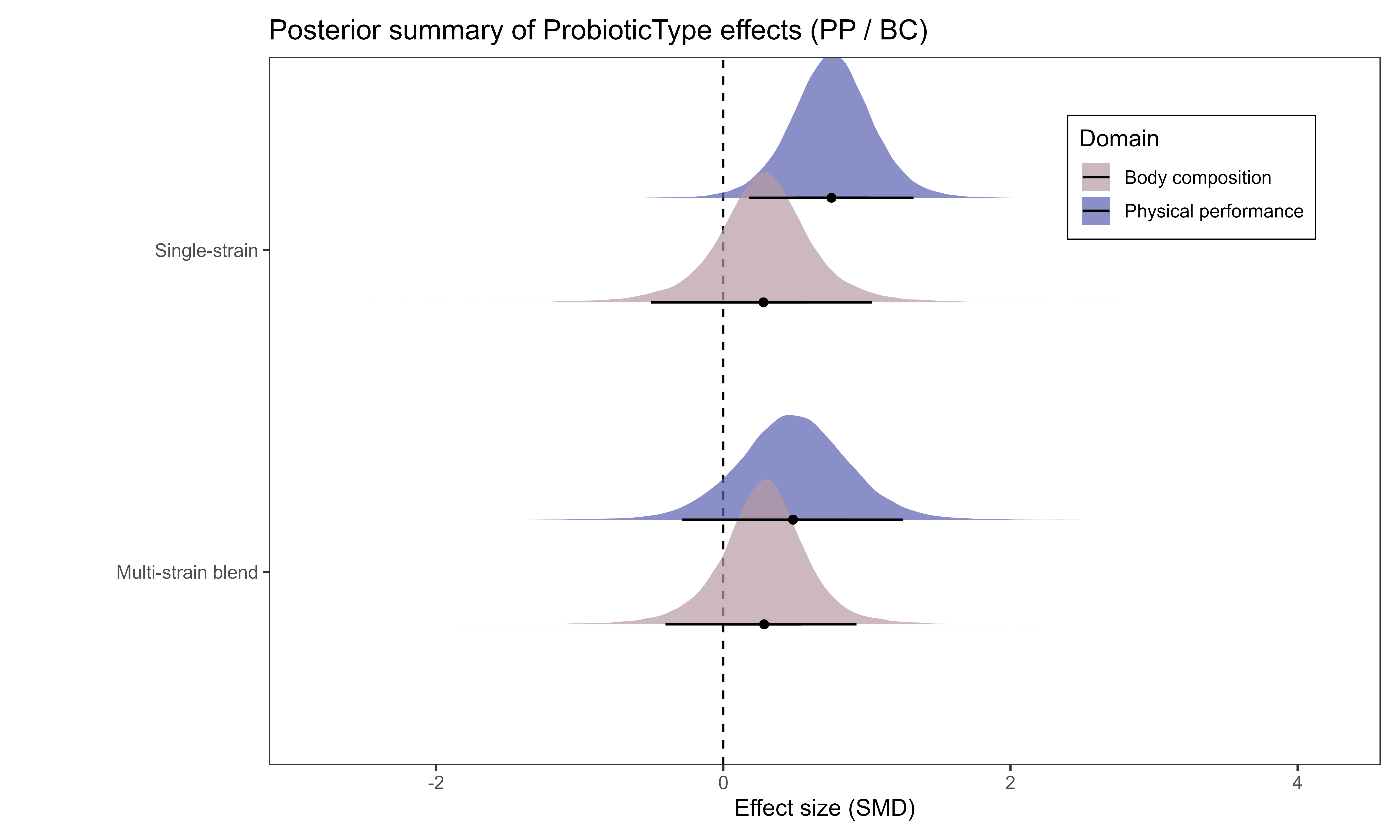


Fig. S7 Bayes factor plot of the Probiotic Preparation Model.


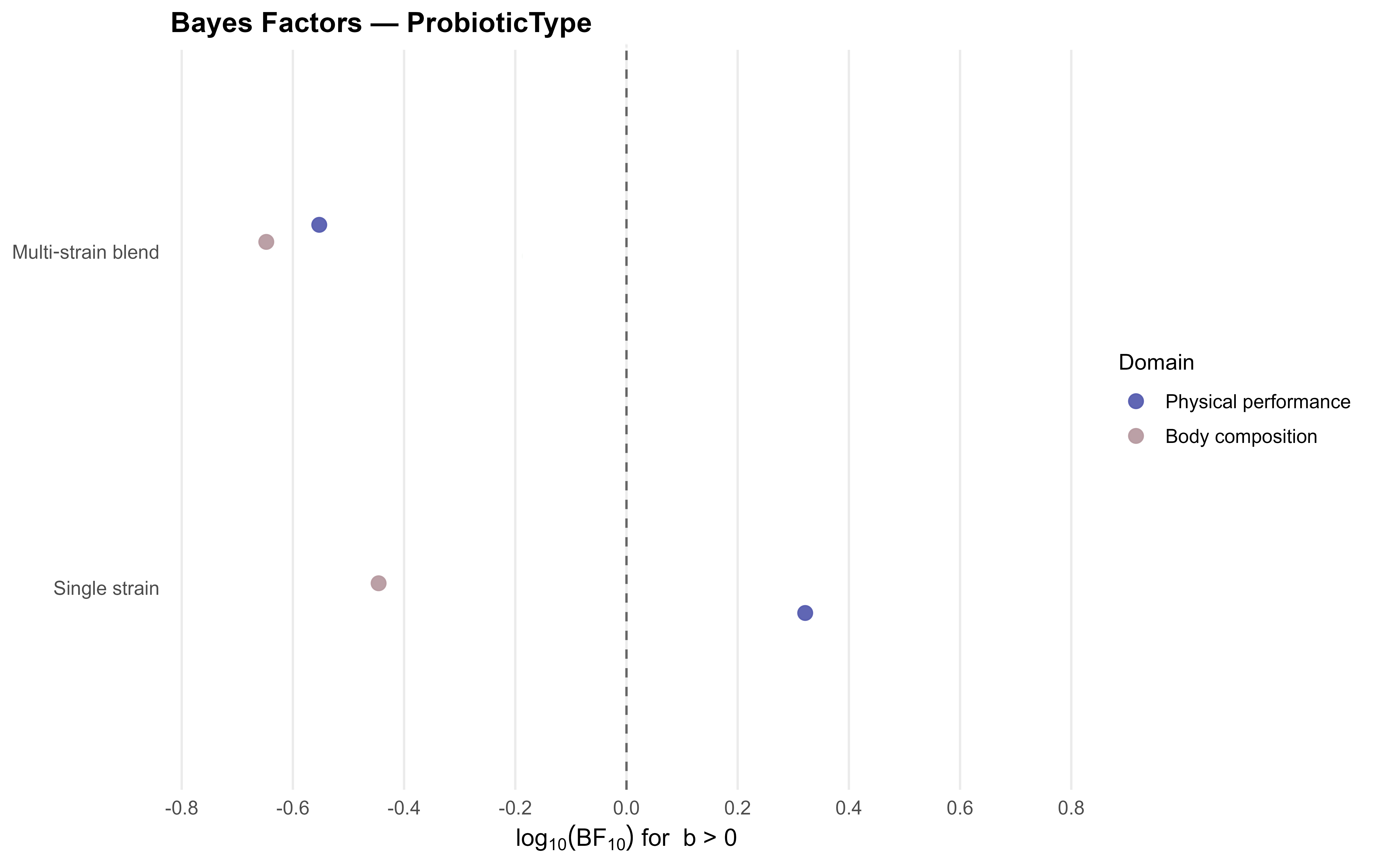


Fig. S8 Posterior summary plot for Probiotic Single-Strain Species Model across physical performance and body composition outcomes.


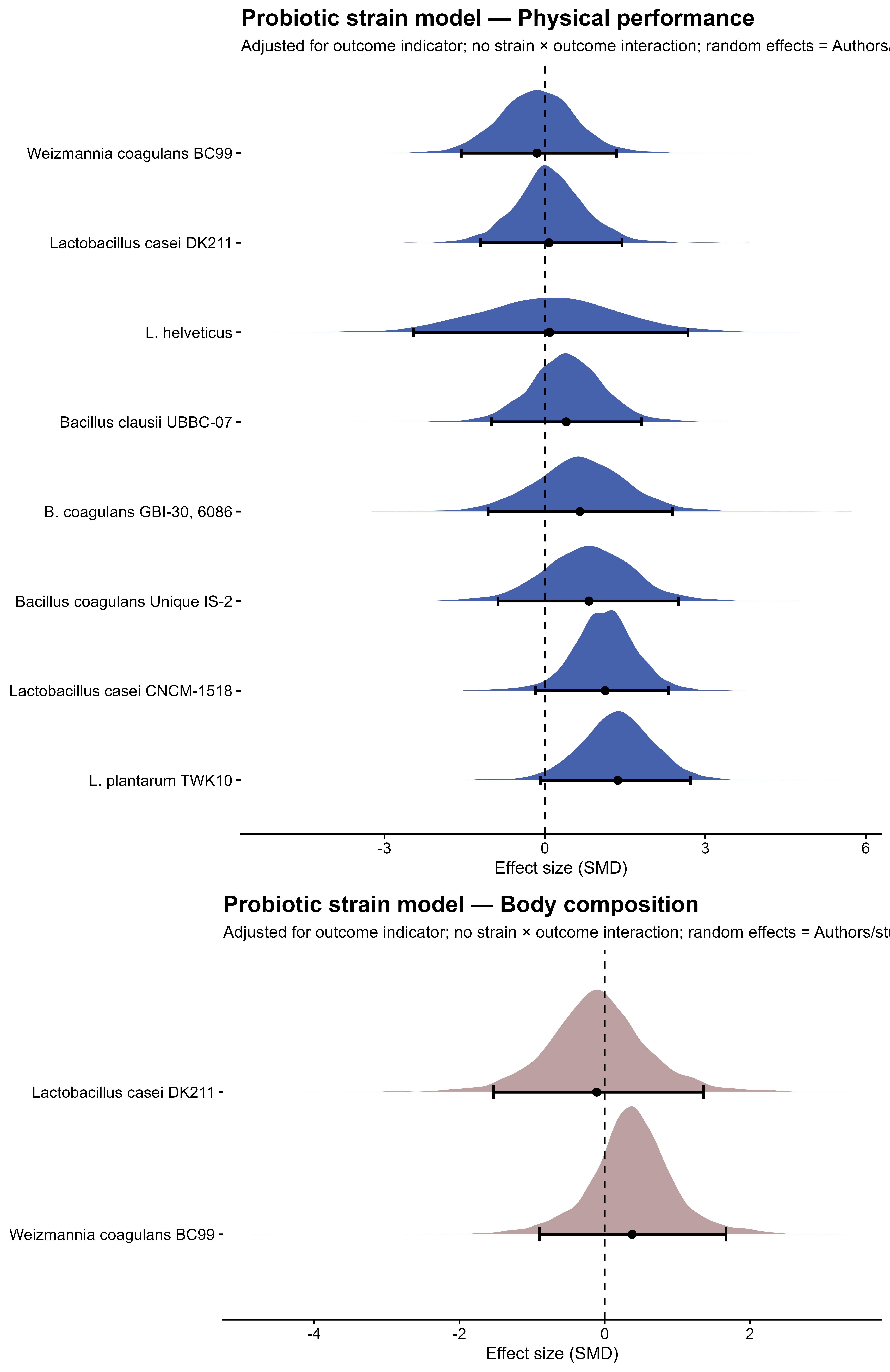


Fig. S9 Bayes factor plot of Probiotic Single-Strain Species Model.


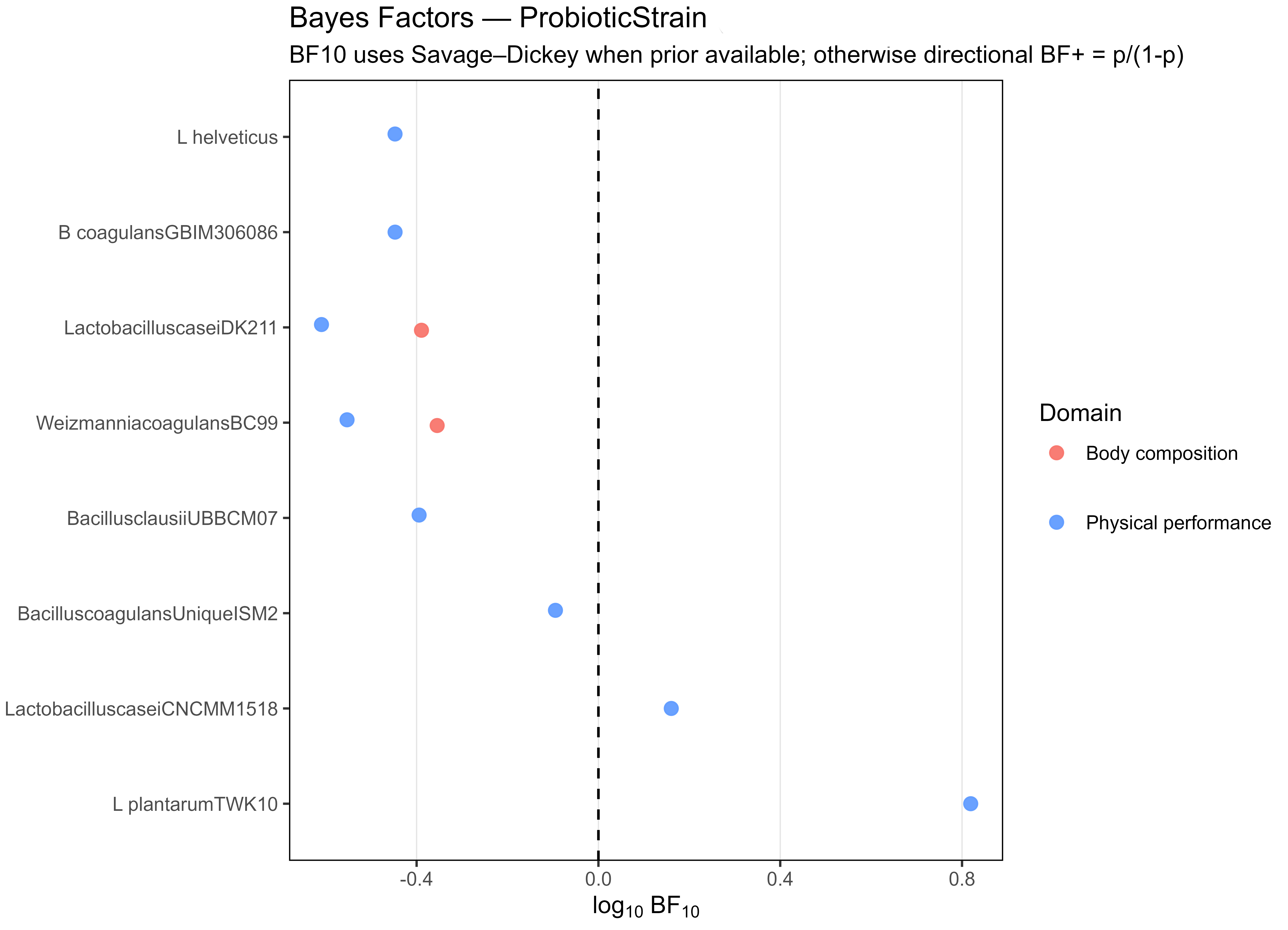


Fig. S10 Posterior summary plot for the Protein Type Model across physical performance and body composition outcomes.


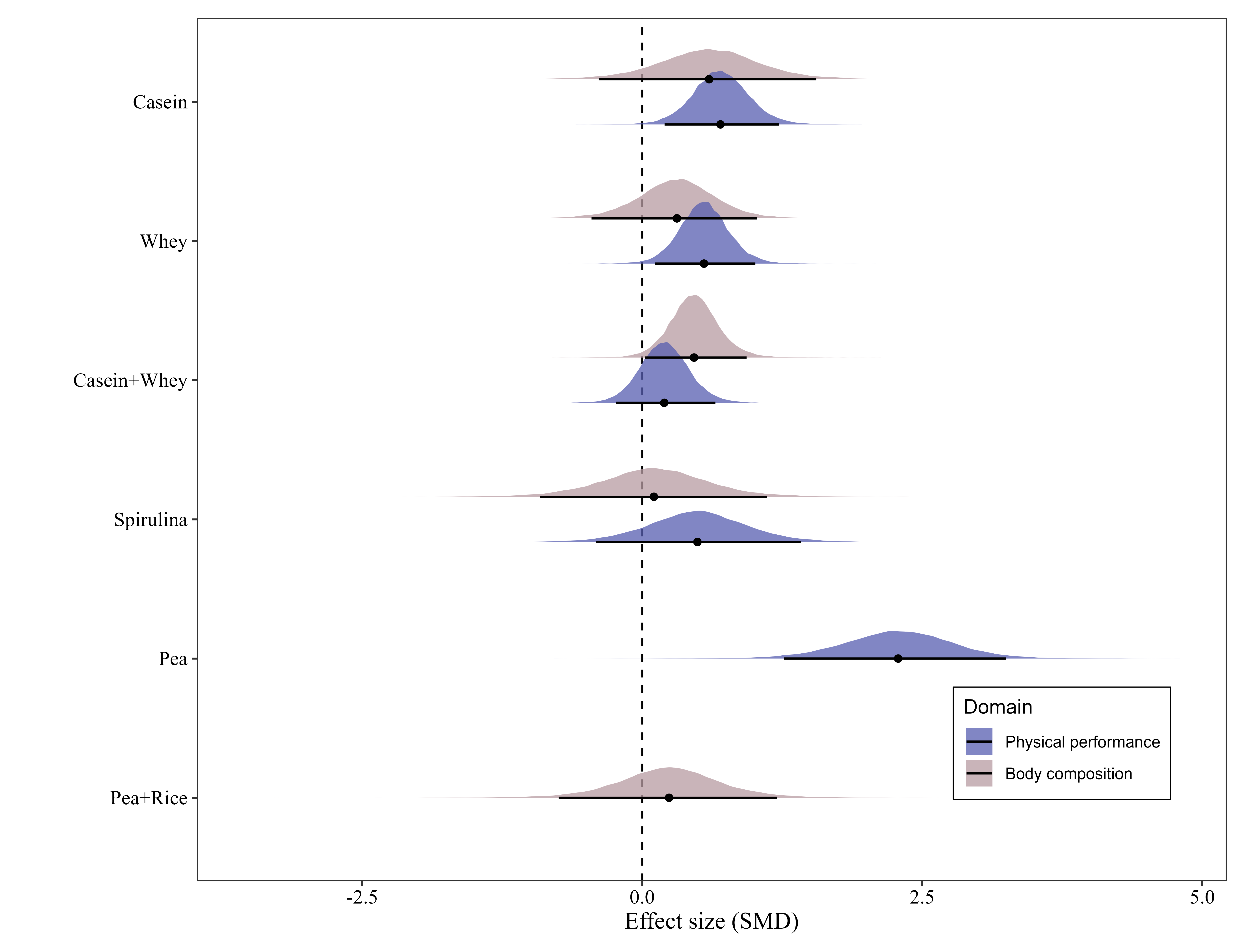


Fig. S11 Bayes factor plot of Protein Type Across Outcome Domains.


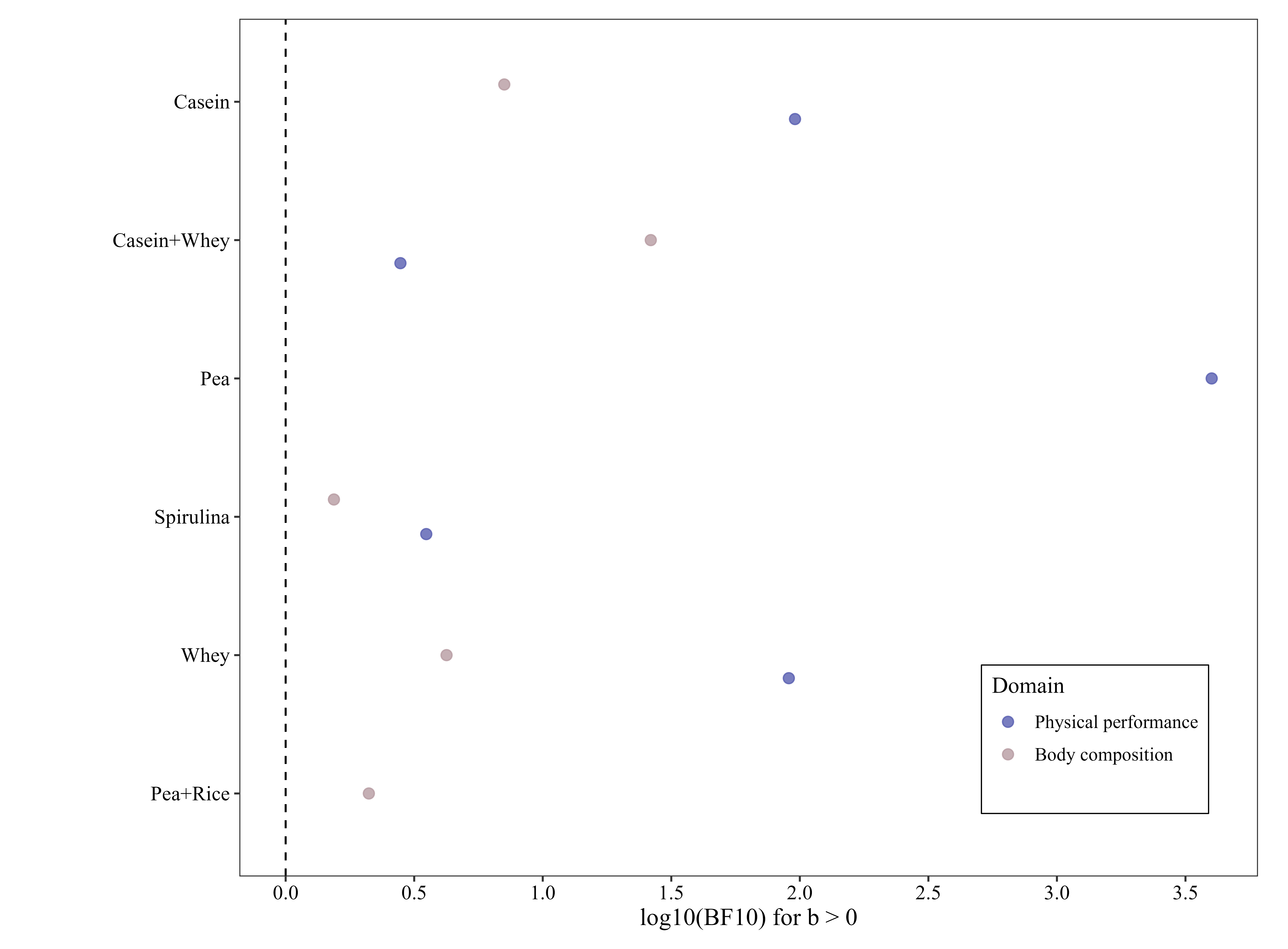


Fig. S12 Posterior summary plot for the Funding Model across physical performance and body composition outcomes.


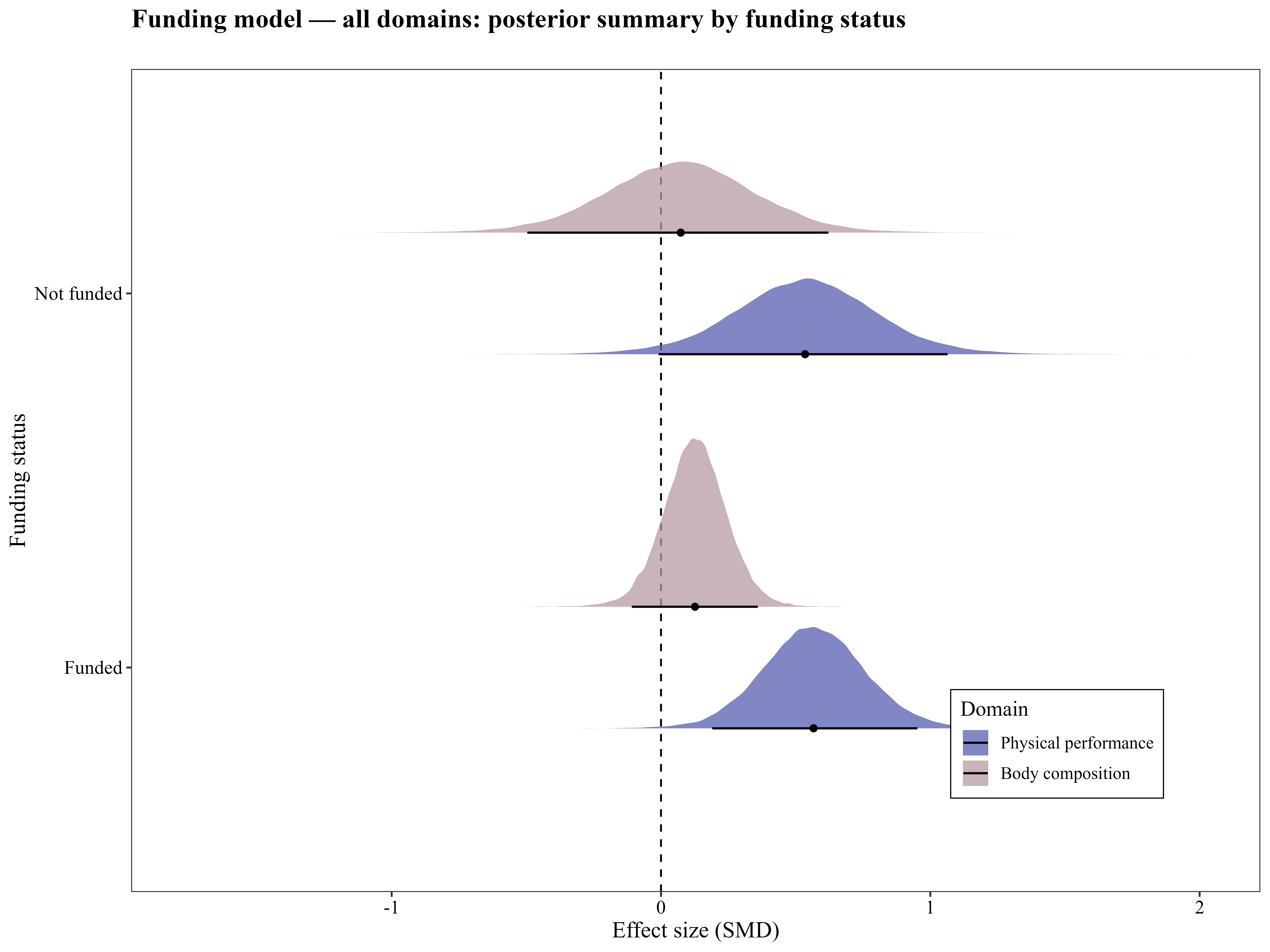


Fig. S13 Bayes factor plot of Funding Model Across Outcome Domains.


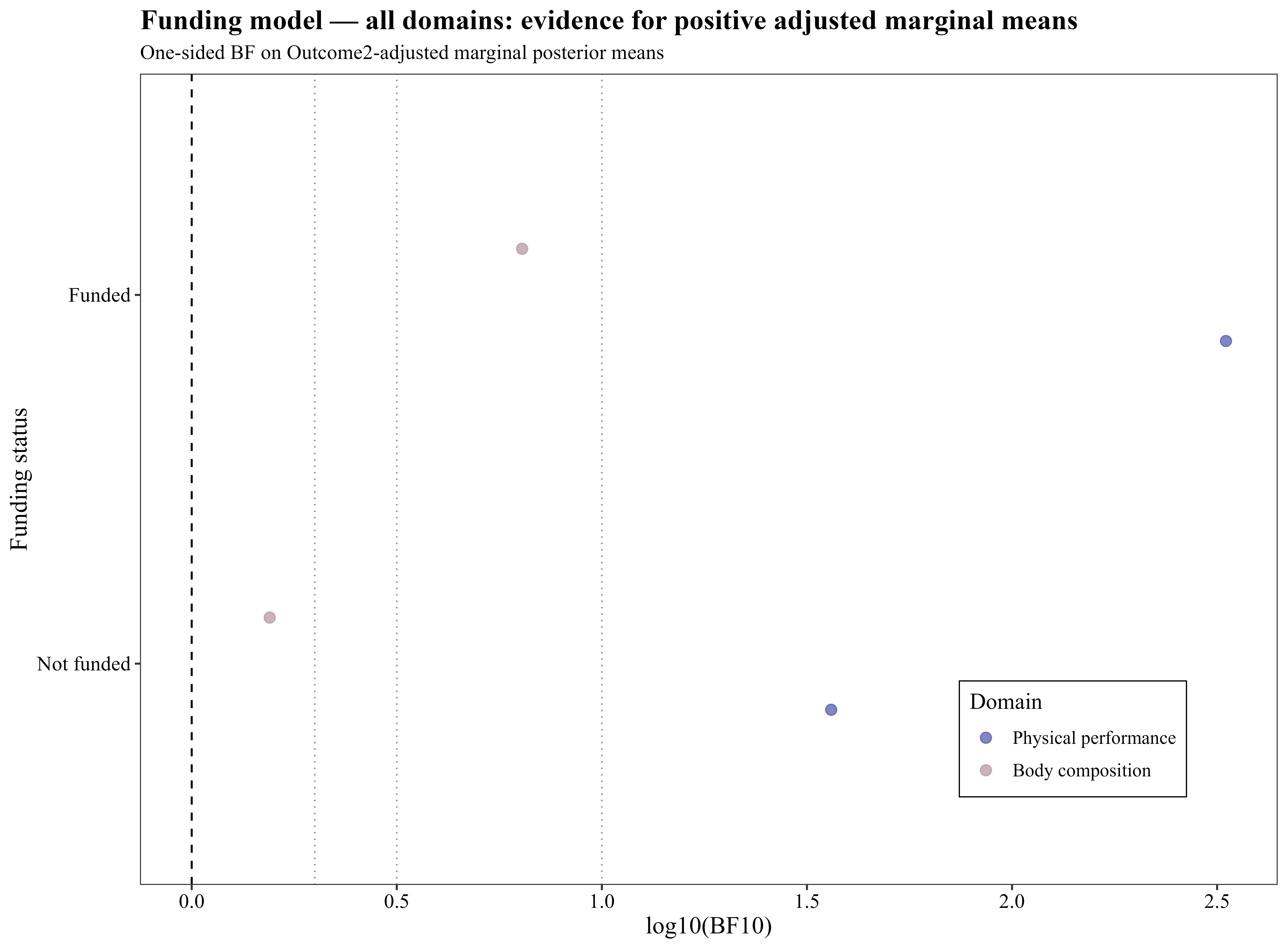

Supplement: Supplementary file 5 [file Table_5.docx]
