# Supplementary figures and images for: Effects of combined protein and probiotic supplementation on physical performance and body composition: a Bayesian multilevel meta-analysis of randomized controlled trials
Source: Front Nutr. 2026 Jun 17;13:1865035. doi: 10.3389/fnut.2026.1865035 (PMC13319104; doi:10.3389/fnut.2026.1865035)

**Supplementary file S8: Graphical Abstract**


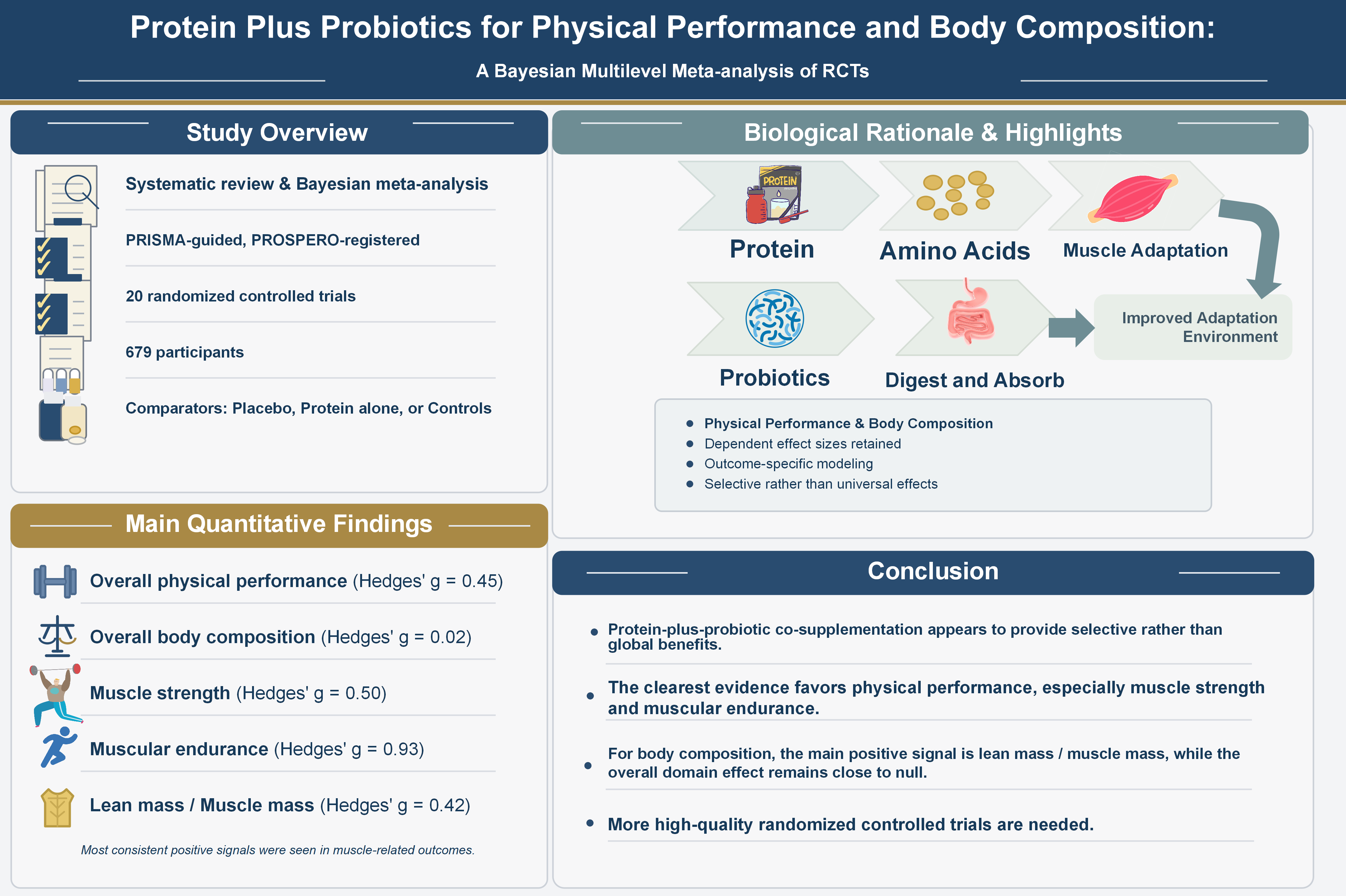


**Fig.S1** Graphical Abstract

Supplement: Supplementary file 8 [file Table_8.DOCX]
